# Molecular mechanisms underpinning transgenerational plasticity in the green sea urchin *Psammechinus miliaris*

**DOI:** 10.1038/s41598-018-37255-6

**Published:** 2019-01-30

**Authors:** Melody S. Clark, Coleen C. Suckling, Alessandro Cavallo, Clara L. Mackenzie, Michael A. S. Thorne, Andrew J. Davies, Lloyd S. Peck

**Affiliations:** 1 British Antarctic Survey, Natural Environment Research Council, High Cross, Madingley Road, Cambridge, CB3 0ET UK; 2 School of Ocean Sciences, Bangor University, Askew Street, Menai Bridge, Anglesey, LL59 5AB UK; 3 Fisheries, Animal and Veterinary Sciences, University of Rhode Island, 4 East Alumni Avenue, Kingston, RI 02881 USA; 4 School of Biological and Marine Sciences, Plymouth University, Drake Circus, Plymouth, PL4 8AA UK; 5 Institute of Aquaculture, University of Stirling, Stirling, FK9 4LA UK; 6 Biological Sciences, University of Rhode Island, 9 East Alumni Avenue, Kingston, RI 02881 USA

## Abstract

The pre-conditioning of adult marine invertebrates to altered conditions, such as low pH, can significantly impact offspring outcomes, a process which is often referred to as transgenerational plasticity (TGP). This study describes for the first time, the gene expression profiles associated with TGP in the green sea urchin *Psammechinus miliaris* and evaluates the transcriptional contribution to larval resilience. RNA-Seq was used to determine how the expression profiles of larvae spawned into low pH from pre-acclimated adults differed to those of larvae produced from adults cultured under ambient pH. The main findings demonstrated that adult conditioning to low pH critically pre-loads the embryonic transcriptional pool with antioxidants to prepare the larvae for the “new” conditions. In addition, the classic cellular stress response, measured via the production of heat shock proteins (the heat shock response (HSR)), was separately evaluated. None of the early stage larvae either spawned in low pH (produced from both ambient and pre-acclimated adults) or subjected to a separate heat shock experiment were able to activate the full HSR as measured in adults, but the capacity to mount an HSR increased as development proceeded. This compromised ability clearly contributes to the vulnerability of early stage larvae to acute environmental challenge.

## Introduction

To make accurate predictions about the resilience of biodiversity in the future, it is essential to understand the cellular mechanisms that underpin species’ responses to change. Evaluations at the molecular level provide *a priori* clues as to how these responses vary between species and suggest who will be the winners and losers under changing conditions^1^. In the marine field, much of the early work manipulating pH and CO_2_ in marine systems, popularly known as Ocean Acidification (OA), has concentrated on early developmental stages (embryos and larvae), as these are potentially the most vulnerable part of the life cycle and key to continued population success^2^. However, many of these early experiments used adults from the wild and spawned them in present day pH sea water. The resulting larvae were then directly decanted into lowered pH sea water and their development monitored. Whilst these types of experimental manipulation mimic the effect of seasonal, episodic high *p*CO_2_ upwelling, such as that observed along the US western coast^3,4^, they do not mirror the real-time trajectory of climate change. These experiments exclude examination of the abilities of organisms to respond via phenotypic plasticity, developmental plasticity and transgenerational plasticity (TGP)^1,5,6^.

An increasing number of studies, many of them in sea urchin species, now show that adult acclimation to low pH prior to spawning can significantly impact larval success^7–10^. This is often called TGP, if both male and female parents are pre-acclimated and maternal conditioning or maternal provisioning if pre-acclimation is restricted to females only^5,6^. In a recent review of TGP in echinoderms and molluscs, the majority of studies reported beneficial effects, although neutral and negative effects were also recorded, with the authors acknowledging that such responses are complex to predict and often time-scale, taxa or species-specific^5^. For example, pre-acclimation of adult sea urchins to low pH, had deleterious effects on the reproductive success of the tropical *Echinometra mathaei*^11^. No effects were observed on the larvae of *Echinometra* species living on venting coral reefs from Papua New Guinea^12^. Mixed effects were discovered in the temperate *Strongylocentrotus droebachiensis*, which showed positive effects on egg size, no effect on female fecundity after prolonged pre-acclimation, but negative effects on F_1_ recruitment and juvenile survival^7^. In both the temperate *Psammechinus miliaris* and Antarctic *Sterechinus neumayeri* positive effects were observed on larval survival at low pH, especially if the whole reproductive cycle was included^9,10^. In a further study on sea urchins and pollution, maternal pre-conditioning of the temperate *Evechinus chloroticus* preloaded the resulting larvae with antioxidants to help mitigate against pollution-induced oxidative damage^13^. To emphasize the complexities of TGP responses, mortality was 100% before the blastula stage in embryos produced from low pH pre-acclimated adults of the cold water sea cucumber (*Cucumaria frondosa*) in the only study to date on lecithotrophic larvae^14^. The picture is even more complicated from a multi-stressor perspective, for example experiments (temperature and low pH) in the bivalves *Mercenaria mercenaria* and *Agropecten irradians* showed negative effects with pre-acclimated adults producing more vulnerable offspring^15^. Similarly studies using the Sydney rock oyster *Saccostrea glomerata* showed positive effects when low pH alone was investigated, but maladaptive responses to multiple stressors^16^. Additionally, the stage at which the embryos or larvae are introduced into low pH is also critical. Sea urchin larvae that were spawned and cultured up to the 4-arm stage in control conditions and then introduced to low pH were more robust than cleaving embryos introduced directly to low pH^17–19^. Thus predicting the outcomes from the experimental manipulation of environmental parameters in marine species is complex, with both parental conditioning and the stage at which the larvae are exposed to the new conditions both critical factors^6^.

In summary, most of the studies on TGP have described the morphological and developmental responses of the F_1_ generation. For cellular mechanisms underpinning these physiological responses, the number of examples is limited. Recent data on larvae spawned from adult populations of *Strongylocentrotus purpuratus* from across a latitudinal pH gradient and also populations pre-acclimated in the laboratory to low pH have demonstrated the upregulation of metabolic pathways redirecting ATP production towards enhancing acid-base homeostasis and ion regulatory capacity, but with potential cellular trade-offs^20,21^. Hence, there is still a significant knowledge gap of not only the differential gene expression underpinning TGP, but also larval robustness through development to altered conditions^5^.

In this study we analysed the gene expression profiles of early stage larvae from the green sea urchin *Psammechinus miliaris* raised under low pH (~1000 µatm). These larvae were produced from either adults raised under ambient conditions (~400 µatm) or pre-acclimated to low pH in IPCC year 2100 conditions (~1000 µatm) to evaluate the cellular level effect of parental conditioning. There was an expectation that larvae produced from adults cultured under ambient conditions and spawned directly into low pH would exhibit the classical stress response to new conditions, namely the heat shock response (HSR). This virtually ubiquitous cellular reaction involves the up-regulation of chaperone proteins, specifically members of the 70 kDa and 90k Da heat shock protein families (Hsp70 and Hsp90). These are highly conserved proteins that deal with the stress-induced denaturation of other cellular proteins^22^. The lack of such a response in the gene expression data was surprising. Therefore the ability of early stage larvae to mount an HSR was further investigated in a separate experiment where both adults and larvae were subjected to an acute heat shock and the resulting expression levels of nine *hsp70* and *hsp90* genes were evaluated by Q-PCR.

## Results

### Gene network analyses in low pH

Expression profiles were produced from cohorts of larvae at 2, 3 and 4 dpf (days post fertilisation) raised in low pH (~1000 µatm). One set of these larvae were produced from adults cultured at ambient pH (~400 µatm) to mimic rapid pH transfer experiments, whilst a second set was produced from low pH acclimated adults to study the effect of parental acclimation. In each case the expression profiles were compared to larvae raised under ambient conditions and produced from adults kept under ambient conditions (controls at ~400 µatm). The number of annotated transcripts which were up-regulated in the treated samples was highly variable (Table 1), but in general, more transcripts at each time point were up-regulated in the samples where both the adults and the larvae were raised in low pH conditions. When these data were entered into the STRING program increasingly complex gene networks were produced over the time course (Supplementary Tables S1–S6).

**Table 1:** Codes and details of larval culturing and comparisons performed in molecular analyses, plus the number of annotated transcripts up-regulated in the treated samples when searched against the (A) GenBank non-redundant and (B) Swissprot human databases.

Time	Treatment		A: GenBank non-redundant	B: Swissprot human
2 days	Ambient adults/low pH larvae	CE2	204	91 (81)
	Low pH for both adults and larvae	E2	274	117 (109)
3 days	Ambient adults/low pH larvae	CE3	92	49 (43)
	Low pH for both adults and larvae	E3	355	153 (138)
4 days	Ambient adults/low pH larvae	CE4	469	315 (309)
	Low pH for both adults and larvae	E4	1356	764 (659)

Unique matches against Swissprot are in brackets. Treatments: In the experimental manipulations the “C” signifies ambient or control conditions of ~400 µatm; the “E” signifies low pH (~1000 µatm, denoted “E”). CE: Adults cultured under ambient conditions with larvae spawned into low pH (samples CE2-CE4); E: larvae spawned into low pH produced from adults also cultured at low pH (samples E2-E4). For the molecular analyses, the transcriptional profiles of all treated larvae were compared against a set of age-matched larvae spawned into ambient conditions produced from ambient cultured adults.

#### Expression profiles for 2dpf larvae

Evaluation of the transcripts up-regulated in 2 dpf larvae produced from ambient adults and raised in low pH showed a small network centred on *tubulin* with connections to other genes involved in microtubule formation and cell division (coded yellow in Supplementary Table S1), indicating that the embryonic cells were actively dividing. A similar network centred on *tubulin* was produced for the genes up-regulated in larvae raised in low pH, produced from adults acclimated at low pH (coded green in Supplemental Table S2). An additional network was also present containing genes involved in glutathione metabolism (*gpx7* and *ggt1*) and inflammation (*mep1B* and *clec4A*) (coded yellow in Supplemental Table S2) indicating the possession of a defence response.

#### Expression profiles for 3dpf larvae

**Figure 1:**
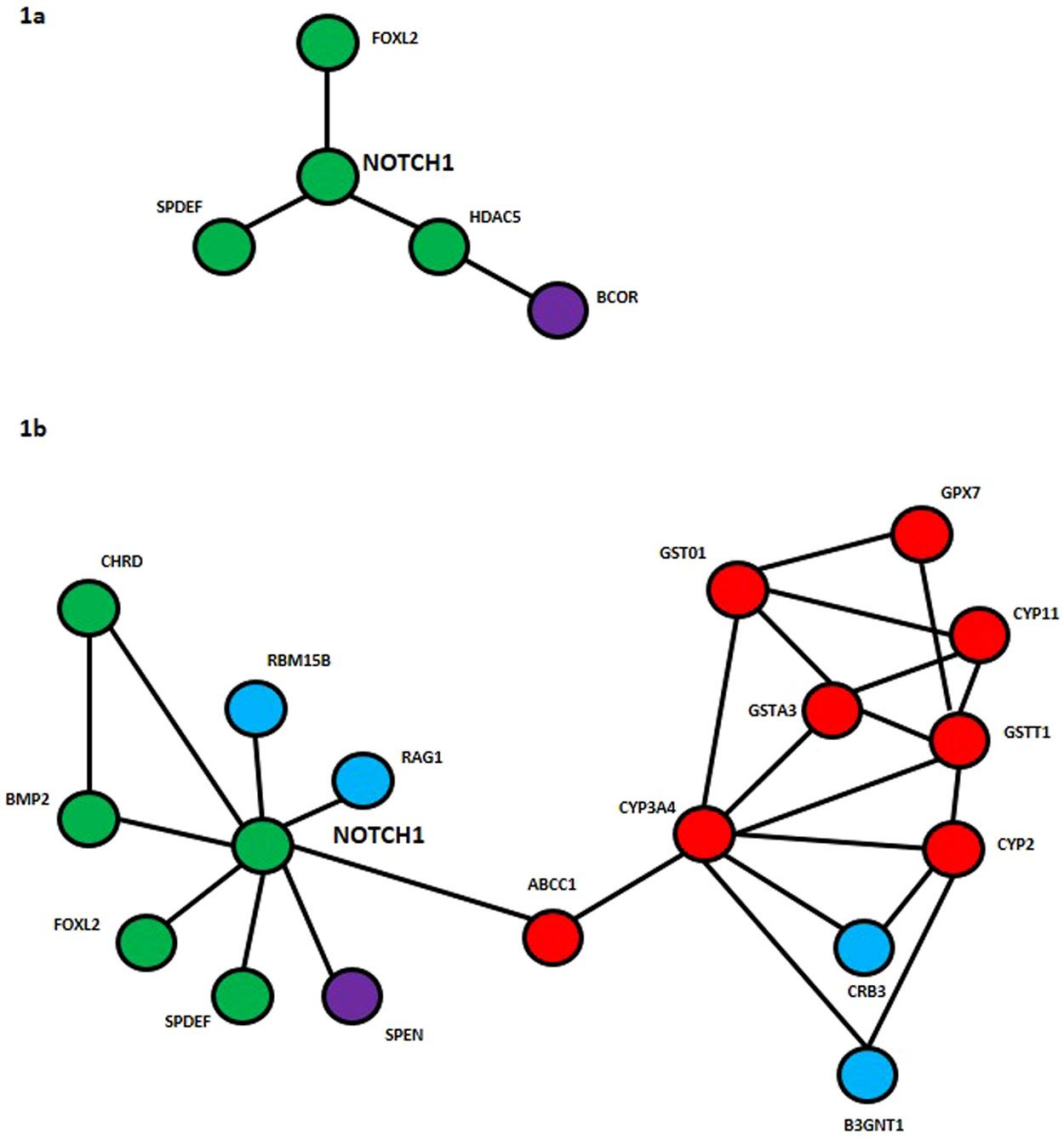
Schematic diagrams of gene network analyses created in STRING for 3dpf larvae detailing putative protein-protein interactions. Protein functions are colour coded: green – signalling proteins and transcription factors; red – antioxidants and detoxification proteins; purple – transcriptional repressors; blue – varied. (**a**) Larvae produced from ambient acclimated adults. Gene names: *notch1*: neurogenic locus notch homolog protein 1; *foxl2*: Forkhead box L2; *hdac5*: Histone deacetylase 2; *spdef*: SAM pointed domain-containing Ets transcription factor; *bcor*: BCL6 corepressor. (**b**) Larvae produced from low pH acclimated adults. Gene names, as for (**a**) plus *gpx7, gst01, gsta3, gstt1*: members of the Glutathione transferase family; *cyp3a4, cyp2, cyp11*: members of the CypP450 family; *abcc1*: Multidrug resistance associated protein 1; *crb3*: Crumbs protein homolog (involved in morphogenesis); *b3gnt1*: N-acetyllactosaminide beta-1,3-N-acetylglucosaminyltransferase (transmembrane protein); *bmp2*: Bone morphogenetic protein; *chrd*: Chordin precursor; *spen*: Msx2-interacting protein; *rag1*: Recombination activating gene (DNA binding protein); *rbm15b*: RNA binding protein 15B (RNA binding).

At this stage larvae in low pH produced from ambient adults produced two networks, one centred on the *notch* gene involving other signalling molecules (Fig. 1a) (coded yellow in Supplementary Table S3) and the second related to RNA processing (coded green in Supplementary Table S3). Three larger inter-connected networks were present among the up-regulated transcripts from the low pH larvae produced from low pH adults. One gene network included the *notch* gene which was connected to other transcriptional activators such as *spdef* and *forkhead* (Fig. 1b) and several members of the antioxidant glutathione-s-transferase family and CypP450 protein family, which is involved in detoxification processes (Fig. 1b) (coded yellow in Supplementary Table S4). A second network (coded green in Supplementary Table S4) was largely concerned with transcriptional regulation, whilst a third (coded blue in Supplementary Table S4) involved metabolic genes. One heat shock protein transcript (contig 7997) with sequence similarity to *hsp701A* was shown as up-regulated (Supplementary Table S4), but was not present in the networks or in the nine *hsp* genes evaluated by Q-PCR.

#### Expression profiles for 4dpf larvae

In this stage, the gene networks in both treatments were far more interconnected and much larger compared to 2 and 3 dpf larvae. In the network produced from up-regulated genes in low pH larvae raised from control pH adults, another important signalling transcript was present (*wnt* (wingless-type MMTV)) and also other transcription factors impacting cell differentiation and growth such as the map kinase *mapk1* and protein kinase (*prkcd*) (Supplementary Table S5). Virtually all of the transcripts up-regulated in the low pH larvae raised from low pH acclimated adults were involved in one large network, in which it was impossible to define specific nodal functional groups. The latter included the signalling genes *notch* and *wnt*, but also the antioxidants; the glutathione-s-transferases, a large number of ribosomal genes and members of the heat shock protein family (contig 35016: *hsp901a1b*; contig 7997: *hsp701a*; contig 18720: *hspa5*; contig 37882: *hspa8*) (Supplementary Table S6). These were small HSP contigs and thus not included in the main HSP listing (Table 2).

**Table 2:** Results of Blast sequence similarity searches indicating putative members of the HSP70 and HSP90 gene families in the *P. miliaris* transcriptome.

Contig identifier	Length (bp)	Blast sequence similarity results					
		Accession no	Species	Description	Score (Bits)	Identity	E
13435	984	D5H3J2	*Psammechinus miliaris*	Putative heat shock protein 70	425.0	66.7	4.6e^−141^
37720	1072	Q06248	*Paracentrotus lividus*	HSP70 IV	674.9	97.5	0.0
44027	1821	A0FKR3	*Lytechinus variegatus*	Mortalin Contains HSP70 domain	987.6	96.0	0.0
46238	665	W4Y6P1	*Strongylocentrotus purpuratus*	Uncharacterised, GN = Sp-Hsp701G Contains HSP70 domain	379.8	85.1	1.7e^−125^
51464	936	W4ZH65	*Strongylocentrotus purpuratus*	Uncharacterised, GN = Sp-Hsp901 Contains HSP90 domain	583.2	87.3	0.0
52063	1583	W4YWW2	*Strongylocentrotus purpuratus*	Uncharacterised, GN = Sp-Hsp902a1 Contains HSP90 domain	902.1	91.9	0.0
62178	1666	W4XYP1	*Strongylocentrotus purpuratus*	Uncharacterised, GN = Sp-Hsp702A Contains HSP70 domain	963.0	95.3	0.0
62830	1682	W4Y0E3	*Strongylocentrotus purpuratus*	Uncharacterised, GN = Hsp701A Contains HSP70 domain	932.6	88.0	0.0
62842	1230	W4Y0E3	*Strongylocentrotus purpuratus*	Uncharacterised, GN = Hsp701A Contains HSP70 domain	680.6	83.4	0.0

All database matches are to sea urchin species.

### Identification of heat shock protein transcripts and transcriptome profiling

Nine contigs were initially identified in the backbone transcriptome, which shared sequence similarity with heat shock proteins (as defined by Blast sequence similarity searching) and also had sufficient overlap in sequence to enable them to be identified as distinct family members (Table 2). Whilst several additional smaller contigs with sequence similarity to *hsp* family members were identified in the transcriptional profiles (Supplementary Tables S1–S6), these sequences were too short to enable a more comprehensive characterisation and thus the list of nine should be considered as a minimal listing of family members present in the genome. Of the nine sequences analysed in more detail, two showed highest sequence similarity to *hsp90* (contig IDs 52063 and 51464) with 48.5% identity and 72.4% similarity at the amino acid level in a 263 amino acid (aa) overlapping region. The first contig (52063) contained the methionine start codon, along with five motifs characteristic of the Hsp90 family (NKEIFLRELISNSSDALDKIR (at aa position 28–48); LGTIAKSGT (aa 95–103); IGQFGVGFYSAYLVAD (aa119–134); IKLYVRRVFI (aa 346–355) and GVVDSEDLPLNISRE (aa 372–386)), but was not full-length, missing the C-terminus with the cellular localisation motif  ^23^. The second contig (51464) was shorter and only encompassed the central portion of the gene, containing only the last two motifs listed above. The remaining seven transcripts were putatively identified as belonging to the *hsp70* family, both on Blast sequence similarity searching and motif searches (Table 2, Supplementary Information S1). Of these it was interesting to note that two (13435, 46238) were the only ones not having a Blast sequence similarity search E value of 0.0, identifying them as more divergent members of this family. A third contig (44027) showed highest sequence similarity to *mortalin*, a gene which, although it has been shown to be non-inducible to heat in vertebrates, has multiple functions including the response to numerous types of environmental stress and chaperone-like activity^24^. The four remaining *hsp70* contigs (37720, 62830, 62842 and 62178) all had the signature for non-organelle family members (comprising *hsp70*, *hsc70* and *grp78*) (Supplementary Information S1). Contig 62178 showed most sequence similarity to *grp78* (Glucose-regulated protein 78 kDa), whilst the remaining three contigs (37720, 62830, 62842) were particularly close in terms of sequence similarity. Analysis of the overlapping 265 amino acid region between these three sequences revealed that the more divergent member of this sub-group (37720) shared most sequence similarity to *hsp70IV* and 86.8–88.7% amino acid similarity to the other two contigs. Contigs 62830 and 62842 matched the same accession number (W4Y0E3: Hsp701A) sharing 84.2% amino acid identity (91.3% similarity). The level of differences between the three transcripts indicated that they were most likely separate isoforms, rather than allelic variants and these data were validated via the Q-PCR analyses.

Interrogation of the OA-generated transcriptome data revealed highly variable expression of the nine different *hsp* transcripts under the different treatments and time points (Table 3). In general the *hsp* transcripts were all poorly expressed in the treated larvae compared with controls. Most of the expression ratios were less than 1 (indicating down regulation) and all were below 2 (the cut-off used in the evaluation of the classical HSR detailed below). The only exception was transcript 37720 (*hsp70IV*) in the CE3 (low pH cultured larvae produced from ambient adults) samples with an expression ratio of 18. Contig 13435 coding for a putative member of the *hsp70* family was not expressed above the analysis threshold in any of the treated samples.

**Table 3:** Ratio of expression levels of the putative HSP70 transcripts in the *P. miliaris* larvae reared under different conditions.

Contig ID	Day 2		Day 3		Day 4	
	CE2	E2	CE3	E3	CE4	E4
13435	—	—	—	—	—	—
37720	—	—	18.00	—	—	—
44027	0.78	—	0.72	—	0.84	0.38
46238	—	0.59	0.28	—	—	0.29
51464	1.39	1.72	—	—	—	0.74
52063	—	1.36	1.50	—	0.90	0.35
62178	0.82	1.13	—	—	—	0.43
62830	0.81	0.82	—	—	1.41	1.46
62842	0.76	1.10	1.96	—	0.72	0.33

Treated larvae were compared with base-line expression levels in larvae raised under ambient conditions generated from ambient cultured adults. Treatments: larvae spawned from ambient cultured adults were directly placed in low pH (CE2-CE4) or larvae spawned from adults cultured in low pH were also spawned into low pH (E2-E4). Dashed line represents the absence of expression.

### Evaluation of the classical heat shock response via heat shock experiments in adults and larvae

**Figure 2:**
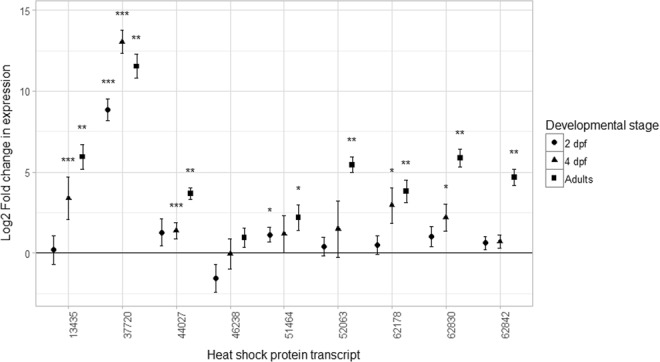
Differential gene expression between heat shock and control conditions for nine *HSP* transcripts in day 2 larvae (n = 3), day 4 larvae (n = 3) and adult *P. miliaris* sea urchins (n = 5). Data are shown as log_2_-transformed fold changes (i.e. −ΔΔCt) ± S.E., calculated according to the 2^−ΔΔCt^ method^69^. Raw Cts (threshold cycles) used in the calculations were first efficiency corrected as follows: Ct ∗ log_2_(E) = E-corrected Ct (where E: amplification efficiency). Asterisks above individual fold change values indicate significantly different expression between heat shock and control treatments (randomisation test performed with REST 2009): *p < 0.05; **p < 0.01; ***p < 0.001. dpf: days post fertilisation.

In a separate *P. miliaris* experiment, adult and 2 dpf and 4 dpf larvae, all cultured and raised in ambient pH, were subjected to an acute heat shock of 25 °C for one hour to evaluate their HSR. All of the nine *hsp70* and *hsp90* gene family members identified from the larval transcriptome were successfully amplified in these samples by standard PCR and Q-PCR. *18s rRNA* was a suitable reference gene as its expression was stable across all samples: Cq values were not significantly different between control and heat shock samples for 2 dpf larvae (t_(3.84)_ = 1.87, p > 0.05), 4 dpf larvae (χ^2^(1) = 0.05, p > 0.05) and adults (t_(17.473)_ = −0.010, p > 0.05). There was an increase in the number of significantly up-regulated *hsp* transcripts in response to heat shock through development: two transcripts were up-regulated in 2 dpf larvae, whilst five were up-regulated in 4 dpf larvae. Eight transcripts were up-regulated in adults (Fig. 2), including one transcript (contig 44027) with highest sequence similarity to *mortalin*, an *hsp70* family member which has not been shown previously to be involved in the heat shock response. Only one transcript (Contig 46238: *hsp701G*) did not show significant differential expression between heat shock and control treatments. Developmental stage also had an effect on the magnitude of up-regulation: the combined log_2_ fold changes in gene expression across all transcripts were significantly different between stages [χ^2^ (2) = 9.358, p < 0.01]. The effect was most evident between 2 dpf larvae and adults (Fig. 2).

## Discussion

These data clearly highlight the molecular mechanisms underpinning TGP in *P. miliaris*, with maternal pre-loading of transcripts involved in the classical stress response, such as antioxidants and chaperone proteins. We suggest that these transcripts play a protective role in the vulnerable early stage larvae until their own transcriptional responses are fully developed. The transcriptional profiling also showed delayed expression of signalling genes, such as *notch* and *wnt*. These transcripts are key regulators of development, interacting with numerous biochemical pathways including cellular homeostasis and calcification, which may explain the morphological differences previously observed in this species, when subjected to low pH experiments^9^ (Supplementary Information S2). There is also a developmental delay in the capacity of *P. miliaris* larvae to express an HSR, the classical response to environmental change, as demonstrated by an additional heat shock experiment and Q-PCR analysis of heat shock proteins (Fig. 2).

One working hypothesis at the start of this study concerned the developmental regulation of the classical environmental stress response, often evaluated as the HSR. It was expected that larvae spawned into low pH from adults held in control conditions would exhibit more of an HSR compared with larvae spawned from adults pre-conditioned to low pH, due to the induction of an acute stress response. In fact it was the low pH larvae produced from low pH acclimated adults that showed expression of a variety of *hsp* transcripts along with genes involved in antioxidant and detoxification activities. These data provide a potential cellular mechanism behind TGP in this species, which results in improved larval performance with adult acclimation to low pH^9^. Current evidence from other species suggests that in general embryos are stable and well buffered from the environment, which is achieved by a process called maternal provisioning or maternal conditioning^25^. Studies, specifically on maternal effects have shown that adults raised in good nutritional conditions can increase the levels of energy sources, such as triglycerides, within the eggs, providing a boost for growth, which can last long after larval feeding has started^26^. Allied to this observation is the demonstration that one of the strategies to increase developmental success is the production of larger eggs^27^ and indeed, this effect has been demonstrated in *P. miliaris* when eggs are produced under altered conditions^9^. Such preparation of the embryo is not restricted to nutritional requirements: in fact, the maternal effect also strongly influences early transcription profiles^21^. The eggs are pre-loaded with maternal transcripts, which include high levels of cellular defence mechanisms, including antioxidants and immune transcripts^25^, the expression of which is triggered on fertilisation and provide the initial development profile of the larvae^28^. Parental conditioning can also influence the response of the offspring, priming them to respond more efficiently to the “new” conditions^21^. This maternal pre-loading of transcripts is evidenced here by the presence of transcripts putatively involved in antioxidant activity and also by the presence of some *hsps* in the low pH larvae produced from low pH pre-treated adults (Supplementary Tables S4 and S6). This indicates the possible protective mechanism behind maternal conditioning of gonads under altered conditions, whereby larvae raised under low pH conditions from acclimated adults can perform at least as well, if not better than larvae raised in control conditions from adults maintained in control conditions^9,10,21,29,30^. Such findings highlight the importance of adult pre-conditioning in a number of urchin species where critical defence mechanisms can be pre-loaded. However there are some species which do not exhibit a favourable TGP to altered conditions^11,14,15^ and others where the initial beneficial effects on the early stage larvae do not continue through to recruitment^7,13^ or diminish when multiple stressors are used^16^. This complexity of response clearly requires further investigation at the molecular level, not only in larvae, but also the adults under pre-acclimation.

It should be noted that even in those cases with beneficial parental acclimation, the maternal transcripts are unstable and are gradually replaced by two waves of minor and major zygotic genome activation as the embryo develops complexity and becomes fully independent^28^. This gradual activation of the zygotic genome is in agreement with the increasing number of transcripts represented through time in the transcriptional profiling detailed here (Table 1) and the heat shock protein Q-PCR results. The lack of any significant up-regulation of heat shock transcripts in the transcription profiles of the larvae raised in low pH (at 2, 3, and 4 dpf), particularly those produced from non-acclimated adults was intriguing. This indicated the potential inability to produce the classic HSR in response to environmental stress, but has been seen previously in other studies^31,32^. In a separate experiment designed to evaluate when *P. miliaris* larvae can mount an HSR, Q-PCR of nine heat shock protein transcripts was used in conjunction with an acute heat shock treatment of both adults and larvae. These demonstrated that the HSR was not fully present in the larvae, but the ability to produce such a response increased in the larvae from 2 dpf to 4 dpf (Fig. 2).

Despite the possession of an active immune system, gastrulation is an extremely sensitive stage of early life history development in sea urchins^34,35^. Previous experiments have shown that the amount of *hsp70* transcript produced at early developmental stages is low and only produced post-blastula^34^. Even with maternal conditioning and pre-loading of transcripts, the levels of *hsp* mRNA are probably insufficient to overcome any cellular damage. It was suggested that this low level of measureable expression was because of the number and size of the cells in the very early embryo. Early embryos have few, large cells, with a low nucleus to cytoplasm ratio, but as the number of cells increase, the cell size decreases and the nucleus to cytoplasm ratio increases. Thus the relative amount of *hsp70* produced per cell becomes increasingly effective at protecting the cell as the number of cells increase^36^. This may be further complicated by selected (and delayed) translational activation of maternal mRNA, as has been shown for *hsp90* or post-translational regulation of *hsp70*^32,33^.

In this study although the embryo stages in this heat shock study were post-blastula, their abilities to produce sufficient *hsps* were still developmentally limited with only two *hsp* transcripts activated in 2 dpf larvae, increasing to four transcripts in 4 dpf larvae. This is much lower when compared with the adults, in which eight *hsps* were expressed in response to an acute heat stress. Additionally the larval transcripts were also expressed at lower levels compared with the adults (Fig. 2), as seen previously in *S. franciscanus*^37^. This may be due to any of the effects discussed above, but also potentially because over-expression of *hsps* can have negative effects on larval development and is thus more tightly regulated during these early stages^38^. The results here are also reflected in recent ocean acidification studies showing the impacts of responses to low pH to be larval stage-dependent^17,18,33,39^. Overall these data indicate the vulnerability of larval stages, but also the need to understand in more detail the transcriptional and translation events in these early life history stages.

In terms of investigating the effects of environmental change on early life history stages, sea urchins are ideal models, as they have a long history in scientific research for deciphering the molecular events involved in the cell cycle and development^40,41^. These studies have identified key regulators of development including *wnt*, *notch* and *β-catenin*. These genes critically initiate the developmental cascades in the urchin. The identification of such key transcripts up-regulated in all the low pH treated larvae, when compared to larvae from control conditions and their presence in enriched gene networks (Fig. 1), implies a delayed expression of these transcripts in this experiment. This may have significant down-stream implications for other biochemical processes, as evidenced by morphological differences between larvae in the different treatments^9,18^. The experiment described here was conducted within the scope of a Masters project (CLM, pers comm; Supplementary Information S2). In the Masters project, the developmental schedule of *P. miliaris* was described along with comprehensive morphometric analyses of larvae raised in ambient and low pH conditions (from ambient acclimated and low pH acclimated adults respectively). These showed altered larval morphometrics with time in the low pH treatment. The larvae raised in low pH had significantly smaller body length-width ratios (F_1,174_ = 8.035, p = 0.005) than counterparts reared under ambient conditions. In addition, larvae raised under low pH had an increased percentage of abnormalities (25–40%) than those raised under ambient conditions. The severity of abnormalities in low pH larvae was more pronounced with missing arms, substantial length differences in arm length, asymmetric body shape etc. compared to ambient pH raised larvae, which had only minor length differences in arm pairs and minor imbalance to body shape (Supplementary Information S2). Changes in larval morphology due to culture in low pH conditions has been documented in virtually every study to date, although there is debate as to the underlying mechanisms. Theories include hypercapnic effects, maintenance costs associated with cellular homoeostasis, reduced capacity for calcification and developmental delay^21,31,42–48^.

Previous transcriptional profiling experiments on sea urchin larvae have shown very variable results, often with considerable down-regulation (in excess of 80%) of transcripts^48^. The effects are often very subtle and thus can be difficult to accurately define (cf. *foxq*2^31^; *wnt8*^49^), particularly if there are consequential trade-off effects^20^. A meta-analysis involving molecular profiling of sea urchin larvae cultured under low pH suggested that shape and size changes in low pH cultured larvae were due to metabolic depression with up-regulation of genes involved in metabolism, ion transport and biomineralization^48^. This meta-analysis did not show over-representation of gene ontologies related to development even though genes annotated to “developmental process” were up-regulated in certain studies^48^. Whilst the most striking finding about the transcriptional profiles described here was the delayed expression of key signalling genes, a number of ion channels and solute carriers were also up-regulated under the different treatments (e.g. *Cacna1a, kcnj18, kcnn3*, solute carriers all denoted *slc* etc) and in general genes involved with skeletogenesis were down-regulated (Supplementary Tables S1–S6). The finding that ion channels and solute carriers were up-regulated in all larvae implies an alteration has occurred in ion homeostasis as a result of culture in low pH. A particularly interesting observation was that more ion channels and solute carriers were up-regulated in the larvae from low pH acclimated adults supporting the results of a previous study which showed that adult conditioning can impact the ion regulatory response of their F_1_^21^ (Supplementary Tables S1–S6). In general genes involved in skeletogenesis were down-regulated in this study (as they were not annotated in the gene lists), the exception was the up-regulation of *bmp*2 and *chrd* in 3 dpf larvae from low pH acclimated adults. These findings, along with altered ion homeostasis may explain why *P. miliaris* larvae cultured in low pH are usually smaller than those cultured in ambient conditions and also the differences in morphological measurements between larvae from different adult pre-treatments^9,18^ (Supplementary Information S2).

Disentangling developmental delay from altered biochemical processes is complex, as the signalling molecules expressed early in development affect multiple biochemical pathways^40,41^. The data here show that the initial disruption to developmental signalling cascades occurs very early in development with the altered expression of the critical transcription factors (*wnt*, *notch* and *β-catenin*) (Fig. 1), which may result in a range of physiological outcomes for the larvae. It is possible be that subtle differences in the expression levels of these initial signalling molecules in different species and their downstream cascades determines the highly variable larval responses, seen in experiments to date. Thus discovery-led RNA-Seq approaches linked to longer and more detailed time course studies may help to dissect out the causality of such species-specific effects and the potential downstream consequences of adult acclimation on later developmental stages such as juveniles and recruitment.

In summary, investigations into larval sensitivities to environmental challenge are revealing the complex nature of the responses, particularly at the molecular level. The data here show that adult *P. miliaris* pre-acclimated to low pH conditions can preload the resulting larvae with protective transcripts such as antioxidants and heat shock proteins, resulting in favourable TGP with larvae better able to survive in the altered conditions. The results of both the transcriptional profiling and the Q-PCR analysis of heat shock proteins clearly show that there is a delay in the ability of larvae to mount a full HSR. This inability (or capacity) of early stage larvae to mount an effective “stress” response clearly contributes to the vulnerability of this early stage of life. Thus maternal conditioning and assessment of the vulnerability of larvae via the characterisation of particularly sensitive stages is important not only for experimental design, but also for modelling where an understanding of recruitment potential underpins future biodiversity predictions.

## Methods

### Animal collection and adult acclimation

*Psammechinus miliaris* sea urchins were collected by scuba divers at 3–10 m in February 2010 from Rubha Garbh, Loch Creran, Scotland. The urchins were transported in aerated cool boxes and transferred into flow-through aquaria at Bangor University’s School of Ocean Sciences, Anglesey. They were then acclimated to ambient (control) pH aquarium conditions (~pH 8) for two months prior to the start of the experiment. Urchins were fed to satiation (approximately 5% mean body weight) twice a week on an artificial diet largely composed of mussels (*Mytilus edulis*) and macroalgae (*Laminaria digitata*). After the two months, animals with a test diameter over 25 mm were chosen as broodstock for the OA trial as this is the size generally considered as reproductively contributing adults in this species^50^. These animals were randomly assigned to experimental tanks in an OA flow-through mesocosm as described previously^18^. Treatments used in this study were derived from the IPCC predictions using the ‘RCP8.5 business-as-usual’ scenario^51^ with parameters calculated and reported following OA community guidelines^52^. Present day control sea water, imported directly from the Menai Straits, measured ~pH_NIST_ 8.06 and *p*CO_2_ calculated at 412 ± 6 µatm (referred to from here on as 400 µatm or ambient), with the year 2100 pH level calculated at ~pH_NIST_ 7.72 and a *p*CO2 of 1013 ± 12 µatm (referred to from here on as 1000 µatm or low pH) (Table 1). Adults were cultured in either 400 µatm or 1000 µatm for a further two months prior to spawning in June of the same year (Supplementary Information S3). No data are available for the water chemistry of Loch Creran, however this experiment resulted in a total of four months culture in water from the Menai Straits prior to spawning, with associated acclimation to water chemistry and diet. This timing is at least as long as previous aquaculture studies in this species, which show full acclimation of *P. miliaris* to manipulation experiments^53,54^. The length of the acclimation period in the new conditions did not cover the whole of the reproductive cycle, as recommended in urchin studies of other species^7,10^. However, in this species previous data have demonstrated that two months is sufficient time to produce significantly improved outcomes for the resulting larvae^9^. Seawater temperatures in holding tanks during acclimation and experimental periods was according to ambient seasonal temperatures of the region (Supplementary Information S3). A 16 L: 8 D photoperiod was used throughout the experiment.

### Spawning and larval culture

Following two months exposure to 400 µatm or 1000 µatm, adult *P. miliaris* from each treatment were induced to spawn at the beginning of July when all urchins, irrespective of treatment, were at stage IV maturity (the normal stage for the peak spawning period)^9,18^. Spawning of adults via injection of 0.5 M KCl and fertilisation of embryos was performed in the same conditions as the parents had been cultured in either ambient or low pH (400 µatm or 1000 µatm) as appropriate, with methodology as described previously^18^. Five females and two males were successfully spawned for each treatment with gametes pooled prior to fertilisation, with the production of three replicates. Embryos and larvae were either cultured under 400 µatm or 1000 µatm conditions, using 1 μm filtered and UV treated seawater obtained from the OA system. 48 hours after fertilisation larval stocking densities were adjusted to one larvae mL^−1^ and a daily feeding regime was started using *Dunaliella tertiolecta* (CCAP 19/6B) cultured in sterile conditions with a daily feeding rate of 1500 cells ml^−1^. 50 mls of larval culture were sampled 2, 3 and 4 days post fertilisation, at which point they were at the feeding pluteus (4 arm) stage. After brief centrifugation at 2,000 rpm to concentrate the larvae, the seawater was carefully removed and the larvae flash frozen in liquid nitrogen and stored at −80 °C until RNA extraction.

### Carbonate chemistry

Daily measurements of temperature and pH were taken using a hand-held temperature compensated pH meter (HI98160 Hanna Instruments UK Ltd). Weekly salinity measurements were made using an ATC portable refractometer calibrated with distilled water prior to use. Total CO_2_ concentration (TCO_2_) was measured twice weekly, in triplicate using a Carbon Dioxide Analyser (Corning 965; Olympic Analytical, UK). The TCO_2_ analyser was blanked to zero and calibrated with Reagecon 2 g L^−1^ CO_2_ standards before each set of readings. Silicate (SiCO_3_) and phosphate (PO_4_) nutrients were determined with a Lachat 8000 two flow injection nutrient analyser in the Scottish Association of Marine Science, Oban, UK. Values for the parameters of total alkalinity (TA), and Ω were calculated with CO2SYS with refitted constants following OA community guidelines^52^ (Table 4).

**Table 4:** Sea water parameters for both of the low pH and heat shock trials.

	Seawater parameter	Control	Altered condition
Low seawater pH trial	pH_NIST_	8.06 ± 0.01	7.72 ± 0.01
	TA (μmol kgSW^−1^)	1921.9 ± 10.1	1937.8 ± 11.8
	pCO_2_ (μatm)	412 ± 6	1013 ± 12
	Ω calcite	2.54 ± 0.03	1.25 ± 0.03
	Ω aragonite	1.63 ± 0.02	0.08 ± 0.02
	Temperature (°C)	14.1 ± 0.1	14.1 ± 0.1
	Salinity	35 ± 0	35 ± 0
Heat shock trial	Temperature (°C)	15.97 ± 0.13	25.41 ± 0.16
	Salinity	35 ± 0	35 ± 0

Directly measured values include pH_NIST_, temperature and salinity with reporting of parameters following OA community guidelines^51^. Heat shock trial data represents the one hour exposure period data only.

### Heat shock experiments

Heat shock experiments were conducted on both larvae and adult *P. miliaris*. The adults were taken from the control system (400 µatm) of the OA experiment described earlier. The control temperature used was 15.8 °C and the heat shock performed at 25 °C for one hour. This temperature was chosen as sufficiently high in relation to the normal temperature range experienced by these urchins to take them over a “stress” threshold and induce a heat shock response^22^. Larvae were produced from broodstock (test diameter (±SE) = 34.87 ± 0.82 mm, whole animal wet mass = 20.50 ± 1.10 g) which were reared under the control conditions described in Table 4. Gametes and subsequent embryos and larvae were maintained at control conditions of 15.8 ± 0.13 °C and salinity 35 on the practical salinity scale until used for the one-hour thermal shock experiments, but otherwise reared using the methods described above. Fertilisation success was 99% showing that gametes were healthy and viable. Heat shocks were performed on larvae cultured under control pH conditions at days two and four dpf, with three replicates for each time period. Prior to the heat shock trial, larvae were collected into a 47 µm mesh and washed directly into the control and higher temperature treatments. After the heat shock of one hour at 25 °C, larvae were centrifuged for two seconds at 7000 rpm, the seawater was removed and the larvae flash frozen in liquid nitrogen and stored at −80 °C until extraction. Two-year old adults (25.5 months) (n = 5) were used in the heat shock experiment. After the adults had been heat shocked, coelomic fluid was removed using a syringe and flash frozen in liquid nitrogen and stored at −80 °C until extraction. Control samples (non-heat shocked) were also taken for both larvae (three replicates) and adults (n = 5).

### RNA extraction and RNA-Seq

RNA was extracted from three replicates of pooled larval samples using TRI reagent (Bioline) and purified on RNeasy mini-columns (Qiagen) according to manufacturer’s instructions. RNA was quantified using either a NanoDrop 1000 photometer (Labtech International, UK) or a Tape Station 2200 (Agilent Technologies). The OA experimental RNAs were subjected to RNA-Seq on an Illumina Hi-Seq (100 bp paired end reads) at The Earlham Institute, Norwich.

### Molecular analyses

In total, 194,339,075 100 bp paired end Illumina reads (C2: 15,601,575; C3: 9,407,731; C4: 15,253,564; CE2: 44,586,274; CE3: 30,382,990; CE4: 14,915,868; E2: 27,799,660;E3: 24,860,228; E4: 11,531,185) were clipped and trimmed for adapter sequence and quality using EA-UTILS^55,56^. The raw Illumina reads were assembled using SOAP denovo (soap.genomics.org.cn) (using a kmer of 91, which produced the greatest number of longer contigs). All kmer contigs greater than 200 bp were run through CD-HIT^57,58^, following which the remaining contigs were run through CAP3^59^. All contigs and singletons greater than 500 bp were then screened for contamination from various sources, including the food (*Dunaliella tertiolecta*) using BLAST and any reads identified as contaminants removed. The remaining sequences were used for both producing the backbone sequence database and also for mapping differential expression patterns. A proportion test was carried out for all comparisons in R^60^ with an adjustment made for multiple testing^61^ with an FDR cutoff set at 0.01. Only mappings where both paired-end reads mapped to the same contig were used to generate expression levels and calculate significance of expression with the following additional criteria for selection and designation of significant change in expression: a fold change greater than 2; an adjusted p value of <0.01; both sets of treatment reads had to have in excess of 10 reads per contig and at least one treatment had to have in excess of 50 reads per contig. Contigs were searched for sequence similarity using BLAST^62^ against the GenBank non-redundant database^63^ with a threshold score of <1e^−10^. Transcripts with sequence similarity to members of the 70 kDa and 90 kDa HSP family were identified from searches of the BLAST data, manually verified and the sequences manipulated, including translation into putative protein sequences, using Bio-Linux^64^. Sequences annotated through GenBank were further searched against the Swissprot databases of different model species. The Swissprot *Homo sapiens* database produced the highest number of matches below 1e^−10^ by at least a factor of 10 compared to the lower vertebrates and invertebrates when searched with the *P. miliaris* data and so it was chosen for further analyses. The Swissprot identifiers were used to input into Panther GO-Slim^65^ and the STRING program, a database of protein-protein interactions (http://string-db.org/), to provide an overview of putative gene networks.

### HSP transcript expression analysis: RNA extraction and reverse transcription

RNA was extracted from three replicates of pooled larval samples using ReliaPrep™ RNA Miniprep Systems (Promega) according to manufacturer’s instructions. The protocol for non-fibrous tissue (tissue input ≤ 5 mg) was followed, with a centrifugation speed of 13,000 × g. RNA from the coelomic fluid of adults was extracted using RNeasy Mini Kit (Qiagen) following the protocol for animal cells, with centrifugation speed of 10,600 × g. Both protocols included a DNase digestion step. Larvae and coelomic fluid were lysed using a TissueRuptor rotor-stator homogeniser (Qiagen), and the final elution volume was 30 µL. RNA concentration and integrity were assessed using a Tape Station 2200 (Agilent Technologies). Complementary DNA (cDNA) was produced from 150 or 500 ng of RNA from adults or larvae respectively, using the QuantiTect® Reverse Transcription Kit (Qiagen) according to manufacturer’s instructions, with a further genomic DNA elimination step.

### Q-PCR primer design and specificity testing

Q-PCR primers for each of the nine HSP transcripts were designed either using the Primer3plus program^66^ or manually in the case of the three very closely related *hsp70* genes (Supplementary Table S7), with primer specificity confirmed by Sanger sequencing of PCR products (performed by Source Bioscience, UK). PCR thermal cycling conditions were as follows: 2 min at 95 °C, 35 cycles of 15 s at 95 °C, 20 s at 60 °C and 30 s at 72 °C. Reactions comprised 1x NH_4_ buffer, 1.6 mM MgCl_2_ solution dNTP mix with 213.9 µM of each dNTP, 1.07 µM of each primer, 0.027 u/µL BIOTAQ™ DNA Polymerase (Bioline) and 3.24 µM bovine serum albumin with 0.5 µL of cDNA.

### Q-PCR

Q-PCR was performed using an Eco Real-Time PCR System (PCR^max^) with the following thermal cycling conditions: 3 min at 95 °C followed by 40 cycles of 5 s at 95 °C and 10 s at 60 °C. Amplification specificity was assessed through melting curve analysis, with an additional step immediately subsequent to the thermal cycling: the products were denatured for 15 s at 95 °C, allowed to re-anneal for 15 s at 55 °C and the temperature was then raised to 95 °C at a ramping rate of 0.25 °C/s. Samples were run in duplicate wells and all assays included no template controls and negative controls (nuclease free water). Amplification efficiencies for each assay were calculated by the EcoStudy software (Illumina) from a standard curve produced through serial dilutions of cDNA template pools (separate pools were prepared from adult and larval samples). The number of dilutions was ≥4 starting from undiluted or 1:2 cDNA for all assays and the dilution factor was either 4 or 10, depending on the assay-specific amplification behaviour. Each q-PCR reaction comprised 1x Brilliant III SYBR® Green master mix (Agilent), 570 nM of each primer, 721 nM bovine serum albumin and 0.5 µL cDNA (total reaction volume: 10.5 µL). Adult sample cDNA was diluted 1:10 for all transcripts except for contigs 46238 and 62830, for which a 1:4 dilution was used. Day 2 and day 4 larval cDNA samples were diluted 1:2 and 1:10 respectively. A *18* *s rRNA* transcript was used as the reference gene for normalisation, after confirming its expression was constant between control and heated samples.

### Q-PCR Data analysis

Amplification efficiency and R^2^ values were obtained using the EcoStudy software (Illumina) with automatic baseline correction and threshold adjustment (Supplementary Table S7). To test for differential expression, each of the transcripts was analysed using REST 2009 Software^67^. The algorithm calculates efficiency corrected relative expression ratios between target and reference genes^68^ for each transcript, and performs a randomisation test producing a P value indicating differential expression between two treatments, with statistical significance for p < 0.05^67^. The default number of randomisations (2000) was used in the analysis. Stable expression of the reference gene used for normalisation (*18s rRNA*) was confirmed by comparing raw Cq values (Supplementary Table S8) between treatments using a Welch’s t-*t*est for day 2 and adult samples, and by a Kruskal-Wallis test for day 4 samples as these did not meet the normality assumption. To test for a difference in the magnitude of differential expression of the HSP transcripts between early developmental stages and adult sea urchins an inter-stage comparison was carried out on the log_2_ fold changes in gene expression across all transcripts (n = 9, see Fig. 2) among day 2 larvae, day 4 larvae and adult sea urchins. Because the data (log_2_ fold changes) were not normally distributed, a Kruskal-Wallis test and a Dunn’s post-hoc test with Holm’s correction for multiple comparisons were performed. All statistical analyses were performed in R^60^.

## Supplementary information

Supplementary information and tables

## Data Availability

All sequence data were submitted to the NCBI Short-Read Archive (SRA) with the accession number: SRP102910 with the separate run accession numbers: C2 SRR5396276; C3 SRR5396277; C4 SRR5396279; CE2 SRR5396289; CE3 SRR5396306; CE4 SRR5396328; E2 SRR5396309; E3 SRR5396331;E4 SRR5396332.
