# Supplementary material for: Molecular mechanisms underpinning transgenerational plasticity in the green sea urchin Psammechinus miliaris
Source: Sci Rep. 2019 Jan 30;9:952. doi: 10.1038/s41598-018-37255-6 (PMC6353892; doi:10.1038/s41598-018-37255-6)
Supplement: Supplementary file 1 — Supplementary information and tables [file 41598_2018_37255_MOESM1_ESM.pdf]

**Supplementary Data**

**Molecular mechanisms underpinning transgenerational plasticity in the green sea urchin *Psammechinus miliaris***

Melody S Clark[1*], Coleen C Suckling[2,3], Alessandro Cavallo[4], Clara L Mackenzie[5], Michael AS Thorne[1], Andrew J Davies[2,6], Lloyd S Peck[1]

[1]British Antarctic Survey, Natural Environment Research Council, High Cross, Madingley Road, Cambridge, CB3 0ET, UK
[2]School of Ocean Sciences, Bangor University, Askew Street, Menai Bridge, Anglesey, LL59 5AB, UK.
[3] Fisheries, Animal and Veterinary Sciences, University of Rhode Island, 134 Woodward Hall, 9 East Alumni Avenue, Kingston, RI 02881.USA.
[4]School of Biological and Marine Sciences, Plymouth University, Drake Circus, Plymouth, PL4 8AA, UK.
[5]Institute of Aquaculture, University of Stirling, Stirling, FK9 4LA, UK.
[6] Biological Sciences, University of Rhode Island, 120 Flagg Road University of Rhode Island Kingston, RI 02881. USA.

[*]Corresponding Author: MS Clark, British Antarctic Survey, Natural Environment Research Council, High Cross, Madingley Road, Cambridge, CB3 0ET, UK. mscl@bas.ac.uk

**Supplementary Information S1:** Assignment of the HSP annotations: HSP90 and HSP70. Alignments of the seven HSP70 gene family members identified from the transcriptome data annotated with domain motifs, with the information also tabulated below.

ClustalW alignments of the seven putative transcripts with sequence similarity to HSP70 gene family members. These are annotated with the following characteristic signature motifs as described in Rensing & Maier (1994): I: [IVL]-D-[LF]-G-T-T-x-S' II: D-[LF]-G(3)-T-F-D, III: [TS]-[VC]-P-A-[YN]-[FY]-N, IV: [NP]-[EG]-P-[TS]-A-A and V: R-A-[RK]-F-E-[ED]-[LM]. Also annotated: A = the hydrophobic linker site between the nuclear binding domain (NBD) and the substrate binding domain (SBD) (Zuiderweg *et al.,* 2013) and two N-linked glycosylation sites (NKSI and NVSA (consensus of N-X-S)) (Munro & Pelham, 1986; Laursen *et al.,* 1997). Although the region where the putative nuclear localisation signal domain is marked (NLS), none of the sequences have sufficient positively charged amino acids in this region to satisfy this motif. Also see Table below for identification of motifs associated with each transcript.

```
Contig13435      1 ------------------------------------------------------------
Contig37720      1 ------------------------------------------------------------
Contig62830      1 ------------------------------------------------------MAKAPA
Contig62842      1 -------------------------------------------------------AKAPA
Contig62178      1 ---------------------------MRKLLMCMCVMALSASLTLADDDDDSKKEVGTV
Contig44027      1 SKNSKNCLRGTSGCVRQISQQRHFSSVLKTSTCSALNSDSRHHQRHPARCYASDTVRGAV
Contig46238      1 ------------------------------------------------------------
consensus        1                                                          .

                         I

Contig13435      1 ------------------------------------------------------------
Contig37720      1 ------------------------------------------------------------
Contig62830      7 VGIDLGTTYSCVGVFQHGKVEIIANDQGNRTTPSYVAFT-DTERLIGDAAKNQTAMNPYN
Contig62842      6 VGIDLGTTYSCVGVFQHGKVEIIANDQGNRTTPSYVAFT-DTERLIGDAAKNQTAMNPSN
Contig62178     34 IGIDLGTTYSCVGVFKNGRVEIIANDQGNRITPSYVAFTSDNERLIGDAAKNQLTSNPEN
Contig44027     61 IGIDLGTTNSCVAIMEGKTPKVLENAEGARTTPSVVAFTKDDERLVGMPAKRQAVTNAKN
Contig46238      1 ------------------------------------------------------------
consensus       61 ........ .....       .. .  . . ... .... . .....  .. .   .  .

Contig13435      1 ------------------------------------------------------------
Contig37720      1 ------------------------------------------------------------
Contig62830     66 TIFDAKRLIGRNFSDAAVQSDMEHWPFEVINDNKKPKIQVEFKGEKKQFYAEEISSMVLL
Contig62842     65 TIFDAKRLMGRNFTDSAVQSDMKHWPFKVVDSSKKPKIEVEFKGEKKQFFAEEISSMVLL
Contig62178     94 TVFDAKRLIGRTWDDSSVQHDLKYFPFKVMNKNSKPHVKVQVEGQDRIFAAEEISAMVLT
Contig44027    121 TLYATKRLIGRRFDDKETQKDIGTVSYEITKASSG---DAWVKANDKVYSPSQVGAFVLT
Contig46238      1 ------------------------------------------------------------
consensus      121 ...  ...... . .   . ..    . .              .  . .    .   ..

                                       III                          IV

Contig13435      1 ------------------------------------------------------------
Contig37720      1 ---------------------PAYFNDAQRQETKDAGVIAGLNVLRIINEPTAAALAYGL
Contig62830    126 KMKETAEAYLGKTVTDAVVTVPAYFNDSQRHATKDAGVISGLNILRIINEPTAAAIAYGL
Contig62842    125 KMKDTAEAYLGKTITDAVVTVPAYFNDSQRHATKDAGIISGLNVLRIINEPTAAAIAYGL
Contig62178    154 KMKETAEAYLGKKVTHAVVTVPAYFNDAQRQATKDAGVIAGLNVMRIINEPTAAAIAYGM
Contig44027    178 KMKETAENYLGTPAKNAVVTVPAYFNDSQRQATKDAGQIAGLNVLRVINEPTAAALAYGM
Contig46238      1 --KEIAEHQCGKETNSTVLTVPVDFSDNQRVLIKKAAEAAGFKILRLISDPSAALLAYQI
consensus      181 ....... ....    ........... .. .............................
```

```
                                   II
                                 ________
Contig13435    1 -------ELYALIFDLGGRTLDVSLMLIEEGIFEIRTSARDTCLGGEDFDNRLVNHLIAE
Contig37720   40 DK-KLTGEKHVLIFDLGGGTFDVSLLAIDDGVFEVLTTAGDTHLGGEDFDNRLVNHLVEE
Contig62830  186 DK-KVGEERNVLIFDLGGGTFDVSVLTIEEGIFEVKSTSGNTHLGGEDFDNRMVKHFITE
Contig62842  185 DK-KVGAERHVLIFDLGGGTFDVSVLTIDDGIFEVRSTSGDTHLGGEDFDNRLVDHFIKE
Contig62178  214 DK-KDG-EKNILVFDLGGGTFDVSLLTIDHGVFEVISTNGDTHLGGEDFDQRVMEHFIKL
Contig44027  238 D---KSGDSVIVVYDLGGGTFDVSVLEIQKGVFEVKSTNGDTFLGGEDFDNMLVNFLVKE
Contig46238   59 GVTKPHDPCNVLVYRLGGSSVSVSVVNVTNGLQRVIASKTSRECAGDDFTKALAVSCAAE
consensus    241 .. .   .. .....***....**.. ...*....... ......*.**..... . . .

                          NLS                                      V
                   _________________                               _
Contig13435   54 FSREHKKDLTTNPVALHRLHTAAEQAKSTLLTSVKAIIKLDSLFEGTD----LDMTVSKA
Contig37720   99 FKRKHKKDMRTNPRAIRRLRTAAERAKRTLSSSAQANIEVDSLFEGID----FYTSISRA
Contig62830  245 FKRKHKKDITANKEQ-QEIEDSLERAKRTLSSSTQASIEIDSLFEGID----YYTSITRA
Contig62842  244 FXEEEQEGHLYQQESSQEIEDSLERAKRTLSSITQASIEIDSLFEGSD----FYTSITRA
Contig62178  272 YKKKKGKDVRKDNRAVQKLRREVEKAKRALSSQHQARIEIESFFDGED----FSETLTRA
Contig44027  295 FQKDSGIDVTKDTMALQRVREAAEKAKIELSSALQTDINLPYLTMDQSGPKHMNLKLSRS
Contig46238  119 FKRQWKMEITDNKRAMGKLYNACETGKHVLSTINSSTISVDSLYEGID----FHSNLTRA
consensus    301 ... ...... .  ...... . *..*..*..  .. *........ .    . ......

                   V
                 _____                                                 *
Contig13435  110 QFEELCSDLFPKCLKPVEKAIRDAKIDKKIIDTVVLVGGSTQIPKIQELLQGFLDGKELN
Contig37720  155 RFEDLCSDLFRKCLEPVERAILDAKMDKKKIDTVVLVGGSTRIPKIQKLLQEFLNGKELN
Contig62830  300 RFEDMNSDLFRGTLEPVEKALSDSKFDKGKIHDLVLVGGSTRIPKIQKLLQDFFKGKELN
Contig62842  300 RFEELCSDLFRGTLAPVEKALNDSKLGKNLIHEIVLVGGSTRIPKIQKLLQDFFNGKELN
Contig62178  328 RFEELNMDLFRSTIKPVQRVMDDAGLKKEEIHEIVLVGGSTRIPKVQQLVKQYFNDKEPS
Contig44027  355 KFESLTESLIKRTIEPCKKAIQDAEINKSDIKEVLLVGGMSRMPKVQSLVQELF-GRQPS
Contig46238  175 KFESIINLPVQRCLQVIGTTLQENNLTAADIQKVIVIGGSSRIPKVQ-------------
consensus    361 .**.. ........ ...... .... .  *......**....**.* ...... .....

                                              A
                                          ________
                 ***
Contig13435  170 KSINPQEVVAYGAAMCAAAVPN-----YEPIVCCDAAPLSLGIETAGGAMTNIIPRYAIV
Contig37720  215 KSINPDEAVAYGAAVQAAILSGDKSDEIKDVLLVDVAPLSLGIETAGGVMSKIIERNTRV
Contig62830  360 KSINPDEAVAYGAAVQAAILSGDKSSEVQDLLLLDVAPLSMGIETAGGVMTALITRNSTI
Contig62842  360 KSINPDEAVAYGAAVQAAILSGDKSEEVQDLLLLDVAPLSMGIETAGGVMT---------
Contig62178  388 RGINPDEAVAYGAAVQGGVLSG--EAETSDLVLLDVNPLTMGIETVGGVMTKLIGRNTVV
Contig44027  414 KSVNPDEAVAVGAAIQGGVLAG----DVTDVLLLDVTPLSLGIETLGGVFTKLINRNTTI
Contig46238      ------------------------------------------------------------
consensus    421 ......................    .. ...................... .. ... .

Contig13435  225 PTRASKTFTTNVDNQPGVSIQVFEGERAMAKDNTRHGHFELTGIPPAPRGVARIEVTFDI
Contig37720  275 PTKASQTFTTYSDNQPGVSIQVFEGERAMTKDNNRLGQFELSGIPPAPRGVPKIEVSFDI
Contig62830  420 PTKQTKTFTTYADNQPGVLIQVFEGERTMTKDNNILGKFELSGIPPAPRGVPQIDVAFDI
Contig62842      ------------------------------------------------------------
Contig62178  446 PTKKSQIFSTAADNQPTVTIQVFEGERSMTKDNHLLGKFDLNGIPPAPRGVPQIEVTFEI
Contig44027  470 PTKKAQVFSTAADGQTQVEIKVCQGEREMAGDNKTLGQFQLVGVPPAPRGVPQIEVTFDI
Contig46238      ------------------------------------------------------------
consensus    481 ... .  ...  .... . ........ . ...  .. ... .......... .......
```

```
                         ****
Contig13435    285 DANGTMTVTAKDDSTGRSEGITFPNEQ-RLSREDIQRMMDDAQR----------
Contig37720    335 DANGIMHVTAKDESSGRSNKIT--------------------------------
Contig62830    480 DANGILNVSAVEKATGKENKITITNDGGRLSKEDIEKMVADAEKFKDEDEAQRE
Contig62842        ------------------------------------------------------
Contig62178    506 DVNGILRVSAEDKGTGNKEKITITNDQNRLTPEDIE------------------
Contig44027    530 DANGIVNVSARDKGTGKEQQIVIQSSG-GLSNDEIENMVRAAEKFAAE------
Contig46238        ------------------------------------------------------
consensus      541 ...... ... .  ...   ..       .. ...
```

Identification of signature motifs in the seven putative transcripts with sequence similarity to HSP70 gene family members. Annotation notes: NBD = nuclear binding domain, SBD = substrate binding domain. None of the sequences contain a putative nuclear localisation signal, which comprises two positively charged amino acids separated by 10-11 amino acids from another block of five, which contains at least three positively charged amino acids.

| **Motif** | **Note on motif** | **Contigs** | | | | | | | **Reference** |
|---|---|---|---|---|---|---|---|---|---|
| | | **13435** | **37720** | **62830** | **62842** | **62178** | **44027** | **46238** | |
| [IVL]-D-[LF]-G-T-T-x-S | Signature I | - | - | √ | √ | √ | √ | - | Rensing & Maier (1994) |
| D-[LF]-G(3)-T-F-D | Signature II | X | √ | √ | √ | √ | √ | X | Rensing & Maier (1994) |
| [TS]-[VC]-P-A-[YN]-[FY]-N | Signature III | - | - | √ | √ | √ | √ | X | Rensing & Maier (1994) |
| [NP]-[EG]-P-[TS]-A-A | Signature IV | - | √ | √ | √ | √ | √ | X | Rensing & Maier (1994) |
| R-A-[RK]-F-E-[ED]-[LM] | Signature V: Characteristic of HSP70, HSC70 and GRP78 | X | √ | √ | √ | √ | X | X | Rensing & Maier (1994) |
| KRK**.KDL***.RAL*R | Putative nuclear localisation signal | X | X | X | X | X | X | X | Rensing & Maier (1994) |
| [DE]-[VIL]-L-LD-V | Hydrophobic linker between NBD & SBD | X | X | √ | √ | √ | √ | - | Zuiderweg *et al.* (2013) |
| NKSI (N-X-S) | N-linked glycosylation site | √ | √ | √ | √ | X | X | - | Munro & Pelham (1986) |
| NVSA (N-X-S) | N-linked glycosylation site | X | X | √ | - | X | √ | - | Munro & Pelham (1986) |


**References:**

Laursen JR, di Liu H, Wu X-J, Yoshino TP (1997) Heat-shock response in a molluscan cell line: Characterisation of the response and cloning of an inducible HSP70 cDNA. *Journal of Invertebrate Pathology* **70,** 226-33.

Munro S & Pelham HRB (1986) An Hsp70-like protein in the ER: Identity with the 78 kd glucose-regulated protein and immunoglobulin heavy chain binding protein. *Cell* **46,** 291-300.

Rensing SA & Maier U-G (1994) Phylogenetic analysis of the stress-70 protein family. Journal of Molecular Evolution. 38, 80-86.

Zuiderweg ERP, Bertelsen EB, Rousaki A, Mayer MP, Gestwicki JE, Ahmad A (2013) Allostery in the Hsp70 chaperone proteins. *Topics in Current Chemistry* **328,** 99-153.

**Supplementary Information S2:** Morphological measurements and abnormality rates in *P. miliaris* larvae cultured under normal and low pH conditions.

These data are taken from a study which was conducted alongside the experiments described in this paper. The data formed part of a Masters report produced by Clara L. Mackenzie entitled "Life in a high $CO_2$ world: The impacts of near-future ocean acidification to the growth, reproductive success and larval recruitment of *Psammechinus miliaris* (Echinodermata: Echinoidea) (2010) University of Bangor. Supervisors A.J. Davies and C.C. Suckling.

The same broodstock and aquarium systems were used as described in this paper, although only control acclimated adults (ambient pH) or two month low pH acclimated adults(End-Century pH) were used to produce larvae, which were then cultured to recruitment. These experiments provided comparative data on larval morphometrics and abnormality rates between the two conditions.

**Larval morphometrics**

Larval morphometrics were conducted at six time points, with measurements taken at each stage as defined below.

**Table 1:** Overview of egg and larval measurements used for comparing effects of pH on larval development.

| DAY | DEVELOPMENTAL STAGE | n | MEASUREMENT |
|---|---|---|---|
| **1** | Egg | 100 | egg diameter |
| **1** | Egg | 50-100 | egg density ($ml^{-1}$) |
| | | | fertilisation success |
| **2** | Blastula/Prism | 30 | hatching success |
| | | | length and width of early larvae |
| **7** | Echinopluteus | 30 | larval length and width, body length and width, stomach length and width, mouth width, post-oral arm length |
| **14, 23, 29** | Echinopluteus | 30 | larval length and width, body length and width, stomach length and width, mouth width, post-oral arm length, rudiment length |

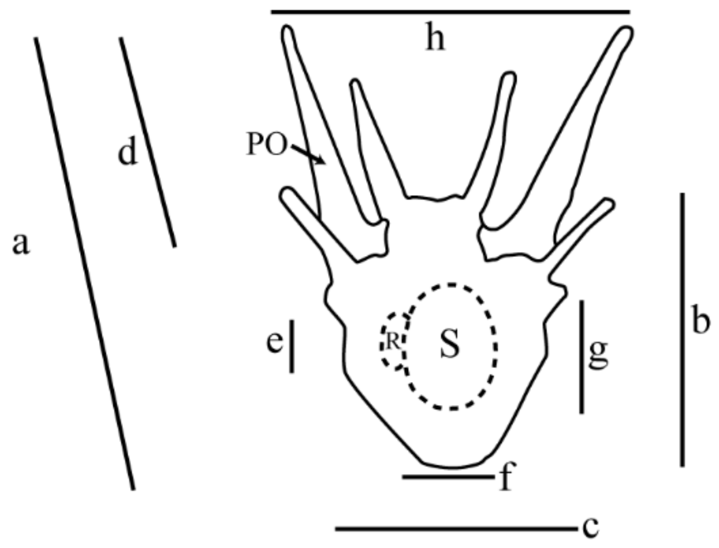


**Figure 1:** Morphometrics of *P. miliaris* larvae: a = total larval length, b = body length, c = body width, d = postoral arm length, e = rudiment length, f = stomach width, g = stomach length, h = total larval width. PO = postoral arm, R = rudiment, S = stomach (adapted from Kelly *et al.*, 2000).

**Results of morphometric measurements**

Only the results of certain time-points are shown to illustrate the points raised in the main discussion, namely the change in larval morphology with treatment over time and abnormality rates.

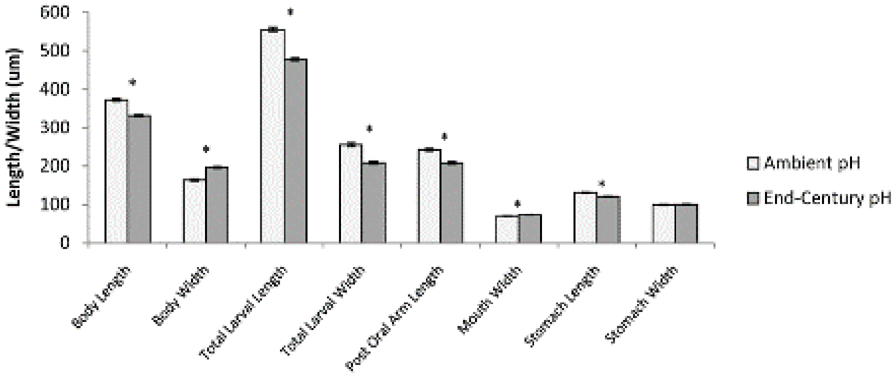


**Figure 2:** Day 7 larval morphometrics of *P. miliaris* larvae under ambient and elevated $CO_2$. * indicates a significant difference between larvae reared under ambient pH and End-century pH. Error bars indicate one standard error of the mean.

Day 7 ambient larvae had significantly greater body length-width ratios ($F_{1,174} = 194.300$, p = < 0.001) and significantly lower larval length-width ratios ($F_{1,174} = 4.375$, p = 0.038) than those reared in high $CO_2$. Stomach length-width ratios did not vary significantly between the two treatment groups.

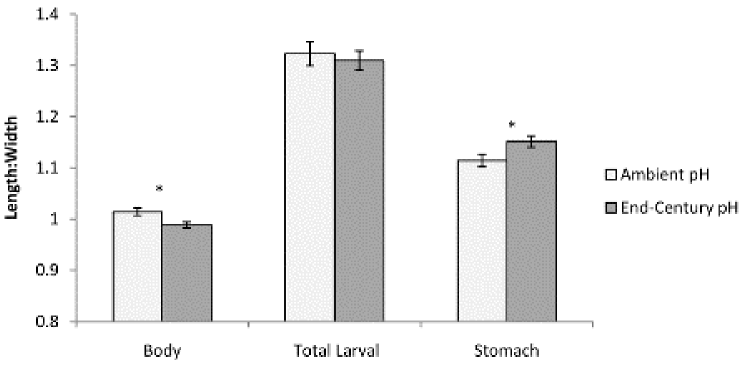


**Figure 3:** Day 23 gross larval morphometrics of *P. miliaris* larvae under ambient and elevated $CO_2$. * indicates a significant difference between larvae reared under ambient pH and End-century pH. Error bars indicate one standard error of the mean.

Day 23 high $CO_2$ larvae had significantly smaller body length-width ratios ($F_{1,174} = 8.035$, $p = 0.005$) but significantly larget stomach length-width ratios ($F_{1,174} = 5.465$, $p = 0.021$) than counterparts reared under ambient conditions. No significant difference was identified in total larval length-width ratios.

**Abnormality rates in culture**

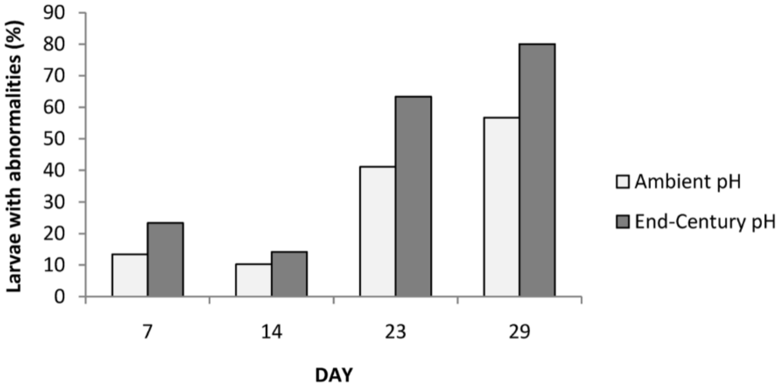


**Figure 4:** Percentage of larvae with abnormalities over larval development (Day) under ambient and End-Century pH conditions.

Larvae raised under End-Century pH conditions had an increased percentage of abnormalities (25-40%) than those raised under ambient conditions. The severity of abnormalities in End-Century conditions was more pronounced with missing arms, substantial length differences in arm length, asymmetric body shape etc. compared to ambient pH raise larvae, which had only minor length differences in arm pairs and minor imbalance to body shape.

**Reference**


Kelly MS, Hunter AJ, Scholfield CL, McKenzie JD (2000) Morphology and survivorship of larval *Psammechinus miliaris* (Gmelin) (Echinodermata: Echinoidea) in response to varying food quantity and quality. Aquaculture, 183, 223-240.

**Supplementary Information S3**

**Urchin culture**

***OA:*** Seawater temperature increased over the course of the adult acclimation period (from 10°C at the beginning of April to 16°C by the beginning of July) as expected with seasonal variability, but temperature compensated $pH_{NIST}$ remained relatively constant for both treatments. Larvae were cultured in July at 16°C. In the End-Century pH trial, temperature and salinity values of seawater were not significantly different, while control pH was significantly higher than the End-Century pH treatment (Kruskal Wallis: pH: $H_{(599)} = 448.50$, $P < 0.001$; One way ANOVA: temperature: $F_{(1,592)} = 0.21$, $P = 0.648$; salinity: $F_{(1,485)} = 1.59$, $P = 0.208$) (Table 1; Supplementary Figure S1 (below)).

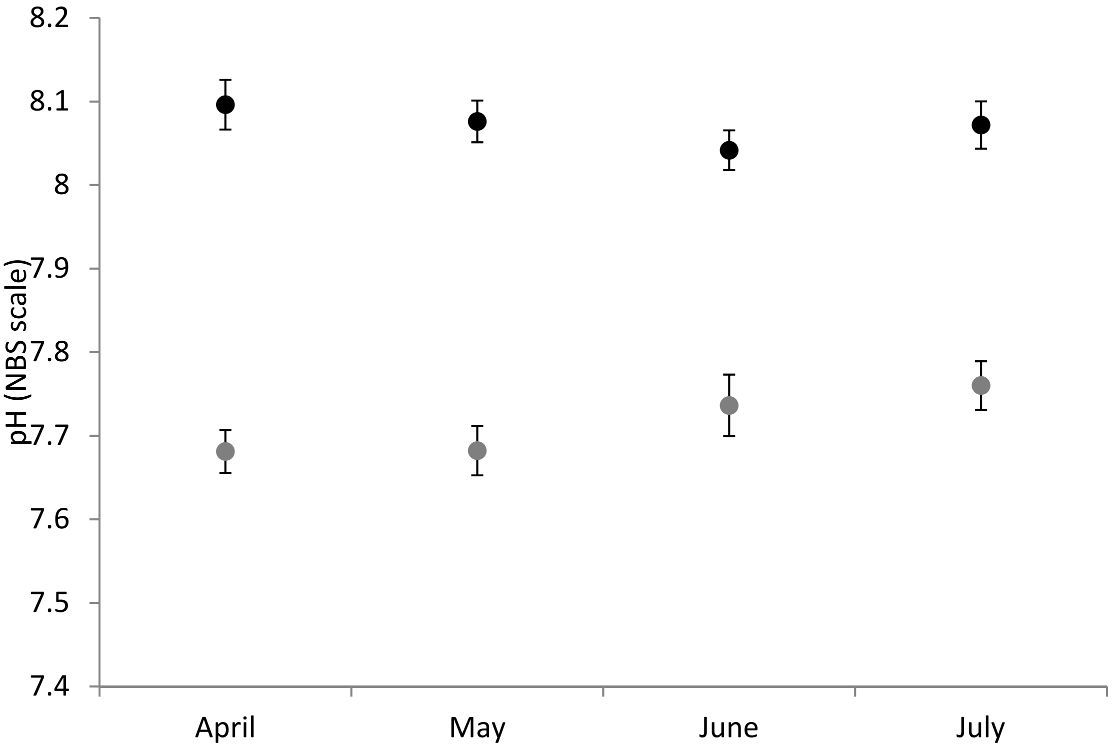


**Supplementary Figure S1:** Mean monthly pH values (± SE) in control (black circles) and low seawater pH (grey circles) trial.

There was no significant difference in the test diameters (TD) of the adult broodstock used in this trial between the treatments (Control (mean TD mm ± SD) 31.03 ± 1.19 and End-Century 32.08 ± 2.06) and there were no mortalities of adults during the two month acclimation period.

***Heat shock trial:*** High temperature treatments were significantly higher than control temperatures (One way ANOVA: $F_{(1,17} = 2148.70$, $P < 0.001$) and salinity values remained the same throughout the experimental period. Adults which were heat shocked were not significantly different in size and mass (Control: TD (± SE) = 25.46 ± 1.58 mm, whole animal wet mass = 8.51 ± 1.54 g; High temperature: TD = 26.14 ± 2.24 mm, whole animal wet mass = 8.80 ± 2.28 g) (One Way ANOVA: TD: $F_{(1,9)} = 0.06$, $P = 0.810$; Wet mass: $F_{(1,9)} = 0.01$, $P = 0.919$).

**Supplementary Table S1:** Putative annotation of transcripts upregulated in CE2 experiment compared to Control samples. Lines annotated in yellow are those involve[illegible] in the one main STRING network identified in these transcripts

| SwissProt ID | Gene Symbol | Gene Name |
|---|---|---|
| Q9NRK6 | ABCB10 | ATP-binding cassette, sub-family B (MDR/TAP), member 1[illegible] |
| P00813 | ada | adenosine deaminase |
| Q9BV57 | ADI1 | acireductone dioxygenase 1 |
| Q8NEM8 | Agbl3 | ATP/GTP binding protein-like 3 |
| Q7Z5J8 | ankar | ankyrin and armadillo repeat containing |
| Q96B67 | arrdc3 | arrestin domain containing 3 |
| Q9H2C2 | ARV1 | ARV1 homolog (S. cerevisiae) |
| Q9Y5Z6 | B3galt1 | UDP-Gal:betaGlcNAc beta 1,3-galactosyltransferase, polypeptide 1 |
| O00555 | Cacna1a | calcium channel, voltage-dependent, P/Q type, alpha 1A subuni[illegible] |
| P62158 | CALM1 | calmodulin 3 (phosphorylase kinase, delta); calmodulin 2 (phosphorylase kinase, delta); calmodulin 1 (phosphorylase kinase, delt[illegible] |
| Q96PB1 | casd1 | CAS1 domain containing 1 |
| P17787 | CHRNB2 | cholinergic receptor, nicotinic, beta 2 (neuronal) |
| Q86X52 | CHSY1 | chondroitin sulfate synthase 1 |
| Q14008 | ckap5 | cytoskeleton associated protein 5 |
| O95406 | CNIH | cornichon homolog (Drosophila) |
| Q86VX2 | commd7 | COMM domain containing 7 |
| Q9UJA2 | CRLS1 | cardiolipin synthase 1 |
| Q9H467 | CUEDC2 | CUE domain containing 2 |
| Q9NXE8 | Cwc25 | coiled-coil domain containing 49 |
| Q9C098 | DCLK3 | doublecortin-like kinase 3 |
| Q8NEL9 | DDHD1 | DDHD domain containing 1 |
| Q8N7U6 | EFHB | EF-hand domain family, member B |
| P11161 | Egr2 | early growth response 2 |
| Q2NKX8 | ERCC6L | excision repair cross-complementing rodent repair deficiency, complementation group 6-lik[illegible] |
| Q99502 | Eya1 | eyes absent homolog 1 (Drosophila) |
| Q9H5I5 | Fam38b | family with sequence similarity 38, member [illegible] |
| Q14320 | Fam50a | family with sequence similarity 50, member [illegible] |
| Q9UJT9 | FBXL7 | F-box and leucine-rich repeat protein 7 |
| Q9UHY8 | fez2 | fasciculation and elongation protein zeta 2 (zygin II) |
| P49326 | FMO5 | flavin containing monooxygenase [illegible] |
| P15408 | Fosl2 | FOS-like antigen 2 |
| Q9BQS8 | Fyco1 | FYVE and coiled-coil domain containing 1 |
| Q9NXP7 | GIN1 | gypsy retrotransposon integrase 1 |
| Q4V328 | Gripap1 | GRIP1 associated protein 1 |
| P63132 | HERVK113 | Endogenous retrovirus group k member 113 polpolyprotei[illegible] |
| Q8IY31 | IFT20 | intraflagellar transport 20 homolog (Chlamydomonas) |
| O94829 | IPO13 | importin 13 |
| Q6DN90 | Iqsec1 | IQ motif and Sec7 domain 1 |
| Q8NAB2 | KBTBD3 | kelch repeat and BTB (POZ) domain containing 3 |
| Q96J84 | KIRREL | kin of IRRE like (Drosophila) |
| Q6ZMI0 | KLRAQ1 | KLRAQ motif containing 1 |
| Q9BRS8 | LARP6 | La ribonucleoprotein domain family, member [illegible] |
| O00370 | LORF2 | LINE-1 retrotransposable element |
| A6PVS8 | Lrriq3 | leucine-rich repeats and IQ motif containing 3 |
| Q16706 | MAN2A1 | mannosidase, alpha, class 2A, member 1 |
| P10636 | mapt | microtubule-associated protein tau |
| Q16820 | mep1b | meprin A, beta |
| Q9UHC1 | MLH3 | mutL homolog 3 (E. coli) |
| Q8N119 | MMP21 | matrix metallopeptidase 21 |
| Q9Y5R2 | mmp24 | matrix metallopeptidase 24 (membrane-inserted) |
| Q9UMX5 | NENF | neuron derived neurotrophic factor |
| O60500 | NPHS1 | nephrosis 1, congenital, Finnish type (nephrin) |
| Q9H668 | OBFC1 | oligonucleotide/oligosaccharide-binding fold containing [illegible] |
| Q9H4Z3 | PCIF1 | PDX1 C-terminal inhibiting factor 1 |
| Q13563 | Pkd2 | polycystic kidney disease 2 (autosomal dominant) |
| O00444 | PLK4 | polo-like kinase 4 (Drosophila) |
| O15162 | plscr1 | phospholipid scramblase 1 |
| Q9ULJ8 | ppp1r9a | protein phosphatase 1, regulatory (inhibitor) subunit 9[illegible] |
| Q9UJF2 | RASAL2 | RAS protein activator like 2 |
| Q9NYR8 | Rdh8 | retinol dehydrogenase 8 (all-trans) |
| Q6NUQ1 | rint1 | RAD50 interactor 1 |
| Q92753 | RORB | RAR-related orphan receptor B |
| A6NKG5 | RTL1 | retrotransposon-like |
| Q96F10 | SAT2 | spermidine/spermine N1-acetyltransferase family member 2 |
| Q9H792 | SGK269 | NKF3 kinase family member |
| Q8WUT9 | Slc25a43 | solute carrier family 25, member 43 |
| Q8N370 | Slc43a2 | solute carrier family 43, member 2 |
| Q8NBI5 | Slc43a3 | solute carrier family 43, member 3 |
| O43246 | slc7a4 | solute carrier family 7 (cationic amino acid transporter, y+ system), member [illegible] |
| Q6X4U4 | SOSTDC1 | sclerostin domain containing 1 |
| Q86XZ4 | Spats2 | spermatogenesis associated, serine-rich 2 |
| P18405 | srd5a1 | steroid-5-alpha-reductase, alpha polypeptide 1 (3-oxo-5 alpha-steroid delta 4-dehydrogenase alpha 1 |
| Q8WXE9 | STON2 | stonin 2 |
| Q9UGT4 | Susd2 | sushi domain containing 2 |
| O75478 | Tada2a | transcriptional adaptor 2 (ADA2 homolog, yeast)-lik[illegible] |
| Q5VWG9 | taf3 | TAF3 RNA polymerase II, TATA box binding protein (TBP)-associated factor, 140kD[illegible] |
| Q9Y6L7 | TLL2 | tolloid-like 2 |
| P68371 | TUBB2C | tubulin, beta 2C |
| P18206 | vcl | vinculin |
| Q9NQW7 | XPNPEP1 | X-prolyl aminopeptidase (aminopeptidase P) 1, solubl[illegible] |
| Q8N883 | ZNF614 | zinc finger protein 614 |
| Q6ZMW2 | znf782 | zinc finger protein 782 |

**Supplementary Table S2:** Putative annotation of transcripts upregulated in E2 experiment compared to control samples. Lines annotated with green and yellow represent those involved in the two main STRING networks identified in these transcripts

| SwissProt ID | Gene Symbol | Gene Name |
|---|---|---|
| Q96QU6 | ACCS | 1-aminocyclopropane-1-carboxylate synthase homolog (Arabidopsis)(non-functional) |
| P22303 | ache | acetylcholinesterase (Yt blood group) |
| Q9H981 | actr8 | ARP8 actin-related protein 8 homolog (yeast) |
| Q86UN6 | Akap14 | A kinase (PRKA) anchor protein 14 |
| Q7Z5J8 | ankar | ankyrin and armadillo repeat containing |
| P15144 | ANPEP | alanyl (membrane) aminopeptidase |
| P55087 | AQP4 | aquaporin 4 |
| P18848 | ATF4C | activating transcription factor 4 (tax-responsive enhancer element B67); activat |
| P06276 | bchE | butyrylcholinesterase |
| Q9BSD7 | C10orf57 | chromosome 10 open reading frame 58; chromosome 10 open reading frame 57 |
| Q9BRX8 | C10orf58 | chromosome 10 open reading frame 58; chromosome 10 open reading frame 57 |
| Q9BTY2 | C1orf57 | chromosome 1 open reading frame 57 |
| Q8NHS4 | C2orf63 | chromosome 2 open reading frame 63 |
| P62158 | CALM3 | calmodulin 3 (phosphorylase kinase, delta); calmodulin 2 (phosphorylase kinase, |
| Q15822 | CHRNA2 | cholinergic receptor, nicotinic, alpha 2 (neuronal) |
| Q9NPF2 | CHST11 | carbohydrate (chondroitin 4) sulfotransferase 11 |
| Q9ULY5 | Clec4e | C-type lectin domain family 4, member E |
| Q9Y6N1 | COX11 | COX11 homolog, cytochrome c oxidase assembly protein (yeast) |
| Q9H467 | CUEDC2 | CUE domain containing 2 |
| Q86UP6 | CUZD1 | CUB and zona pellucida-like domains 1 |
| Q9NXE8 | Cwc25 | coiled-coil domain containing 49 |
| P08684 | CYP3A4 | cytochrome P450, family 3, subfamily A, polypeptide 4 |
| Q5H9S7 | DCAF17 | chromosome 2 open reading frame 37 |
| Q9C098 | DCLK3 | doublecortin-like kinase 3 |
| Q99489 | DDO | D-aspartate oxidase |
| Q9NUL7 | Ddx28 | DEAD (Asp-Glu-Ala-Asp) box polypeptide 28 |
| Q14147 | DHX34 | DEAH (Asp-Glu-Ala-His) box polypeptide 34 |
| Q9UGM3 | DMBT1 | deleted in malignant brain tumors 1 |
| O43854 | edil3 | EGF-like repeats and discoidin I-like domains 3 |
| P11161 | Egr2 | early growth response 2 |
| Q9BY44 | Eif2a | eukaryotic translation initiation factor 2A, 65kDa |
| P21128 | Endou | 26 serine protease |
| Q6UWV6 | ENPP7 | ectonucleotide pyrophosphatase/phosphodiesterase 7 |
| Q2NKX8 | ERCC6L | excision repair cross-complementing rodent repair deficiency, complementation gr |
| O60427 | Fads1 | fatty acid desaturase 1 |
| Q9UBU6 | FAM8A1 | family with sequence similarity 8, member A1 |
| Q9NW38 | fancl | Fanconi anemia, complementation group L |
| P57775 | FBXW4 | F-box and WD repeat domain containing 4 |
| Q8N8Q3 | flj35220 | hypothetical protein FLJ35220 |
| P49326 | FMO5 | flavin containing monooxygenase 5 |
| P58012 | Foxl2 | forkhead box L2 |
| Q9BX68 | FUCA2 | fucosidase, alpha-L- 2, plasma |
| Q11128 | FUT5 | fucosyltransferase 5 (alpha (1,3) fucosyltransferase) |
| P51993 | FUT6 | fucosyltransferase 6 (alpha (1,3) fucosyltransferase) |
| P50440 | gatm | glycine amidinotransferase (L-arginine:glycine amidinotransferase) |
| P04062 | GBA | glucosidase, beta; acid (includes glucosylceramidase) |
| P19440 | ggt1 | gamma-glutamyltransferase light chain 3; gamma-glutamyltransferase 4 pseudogene; |
| Q8N2G8 | GHDC | GH3 domain containing |
| Q9NXP7 | GIN1 | gypsy retrotransposon integrase 1 |
| Q96SL4 | GPX7 | glutathione peroxidase 7 |
| P42262 | GRIA2 | glutamate receptor, ionotropic, AMPA 2 |
| Q13889 | gtf2h3 | general transcription factor IIH, polypeptide 3, 34kDa |
| P84243 | h3f3a | H3 histone, family 3B (H3.3B); H3 histone, family 3A pseudogene; H3 histone, fam |
| Q9UJM8 | HAO1 | hydroxyacid oxidase (glycolate oxidase) 1 |
| Q96MB7 | HARBI1 | harbinger transposase derived 1 |
| Q9BY42 | HINT2 | histidine triad nucleotide binding protein 2 |
| Q7LGA3 | Hs2st1 | heparan sulfate 2-O-sulfotransferase 1 |
| P22304 | ids | iduronate 2-sulfatase |
| Q8WV93 | LACE1 | lactation elevated 1 |

| | | |
|---|---|---|
| A6PVS8 | Lrriq3 | leucine-rich repeats and IQ motif containing 3 |
| Q16820 | mep1b | meprin A, beta |
| Q6ZSS7 | MFSD6 | major facilitator superfamily domain containing 6 |
| Q96AX9 | mib2 | mindbomb homolog 2 (Drosophila) |
| O75431 | MTX2 | metaxin 2 |
| Q8WXR4 | MYO3B | myosin IIIB |
| Q9Y3Q0 | NAALAD2 | N-acetylated alpha-linked acidic dipeptidase 2 |
| Q8NBP7 | pcsk9 | proprotein convertase subtilisin/kexin type 9 |
| Q13608 | PEX6 | peroxisomal biogenesis factor 6 |
| O75928 | PIAS2 | protein inhibitor of activated STAT, 2 |
| O00625 | PIR | pirin (iron-binding nuclear protein) |
| Q13563 | Pkd2 | polycystic kidney disease 2 (autosomal dominant) |
| Q63HM9 | PLCXD3 | phosphatidylinositol-specific phospholipase C, X domain containing 3 |
| Q9P215 | Pogk | pogo transposable element with KRAB domain |
| Q9H1D9 | Polr3f | polymerase (RNA) III (DNA directed) polypeptide F, 39 kDa |
| P15918 | RAG1 | recombination activating gene 1 |
| Q8NHQ8 | rassf8 | Ras association (RalGDS/AF-6) domain family (N-terminal) member 8 |
| P78509 | RELN | reelin |
| O00370 | RNASET2 | ribonuclease T2 |
| O00584 | RNASET2 | ribonuclease T2 |
| Q92753 | RORB | RAR-related orphan receptor B |
| P46777 | rpl5 | ribosomal protein L5 pseudogene 34; ribosomal protein L5 pseudogene 1; ribosomal |
| P04843 | RPN1 | ribophorin I |
| A6NKG5 | RTL1 | retrotransposon-like |
| Q9HBX9 | RXFP1 | relaxin/insulin-like family peptide receptor 1 |
| Q8WTV0 | SCARB1 | scavenger receptor class B, member 1 |
| Q9HD40 | sepsecs | Sep (O-phosphoserine) tRNA:Sec (selenocysteine) tRNA synthase |
| Q9H173 | SIL1 | SIL1 homolog, endoplasmic reticulum chaperone (S. cerevisiae) |
| Q12908 | SLC10A2 | solute carrier family 10 (sodium/bile acid cotransporter family), member 2 |
| Q9NY64 | SLC2A8 | solute carrier family 2 (facilitated glucose transporter), member 8 |
| Q07837 | Slc3a1 | solute carrier family 3 (cystine, dibasic and neutral amino acid transporters, a |
| Q8NBI5 | Slc43a3 | solute carrier family 43, member 3 |
| Q9GZV3 | SLC5A7 | solute carrier family 5 (choline transporter), member 7 |
| O95238 | SPDEF | SAM pointed domain containing ets transcription factor |
| P30874 | Sstr2 | somatostatin receptor 2 |
| O75478 | Tada2a | transcriptional adaptor 2 (ADA2 homolog, yeast)-like |
| Q5VWG9 | taf3 | TAF3 RNA polymerase II, TATA box binding protein (TBP)-associated factor, 140kDa |
| Q8WUH2 | TGFBRAP1 | transforming growth factor, beta receptor associated protein 1 |
| Q3ZCQ8 | timm50 | translocase of inner mitochondrial membrane 50 homolog (S. cerevisiae) |
| O43897 | TLL1 | tolloid-like 1 |
| Q9Y6L7 | TLL2 | tolloid-like 2 |
| Q53FA7 | TP53I3 | tumor protein p53 inducible protein 3 |
| Q13748 | TUBA3D | tubulin, alpha 3d; tubulin, alpha 3c |
| P68371 | TUBB2C | tubulin, beta 2C |
| Q8TDH9 | TXNDC5 | thioredoxin domain containing 5 (endoplasmic reticulum); muted homolog (mouse) |
| P19971 | Tymp | thymidine phosphorylase |
| Q8N6F8 | wbscr27 | Williams Beuren syndrome chromosome region 27 |
| P47989 | XDH | xanthine dehydrogenase |
| Q9NQW7 | XPNPEP1 | X-prolyl aminopeptidase (aminopeptidase P) 1, soluble |
| Q8TBF4 | ZCRB1 | zinc finger CCHC-type and RNA binding motif 1 |
| Q6ZMW2 | znf782 | zinc finger protein 782 |

**Supplementary Table S3:** Putative annotation of transcripts upregulated in CE3 experiment compared to control samples. Lines annotated with annotated with green and yellow represent those involved in the two main STRING networks identified in these transcripts

| SwissProt ID | Gene Symbol | Gene Name |
|---|---|---|
| P12821 | ACE | angiotensin I converting enzyme (peptidyl-dipeptidase A) 1 |
| Q09666 | ahnak | AHNAK nucleoprotein |
| Q9NRL2 | BAZ1A | bromodomain adjacent to zinc finger domain, 1A |
| Q6W2J9 | bcor | BCL6 co-repressor |
| Q5VT06 | CEP350 | centrosomal protein 350kDa |
| Q12996 | CSTF3 | cleavage stimulation factor, 3' pre-RNA, subunit 3, 77kDa |
| Q7Z449 | CYP2U1 | cytochrome P450, family 2, subfamily U, polypeptide 1 |
| Q9Y2E4 | dip2c | DIP2 disco-interacting protein 2 homolog C (Drosophila) |
| Q8WXX0 | DNAH7 | dynein, axonemal, heavy chain 7 |
| Q92874 | DNASE1L2 | deoxyribonuclease I-like 2 |
| Q9BQC3 | DPH2 | DPH2 homolog (S. cerevisiae) |
| Q9C0D9 | Ept1 | selenoprotein I |
| Q8IUD2 | ERC1 | ELKS/RAB6-interacting/CAST family member 1 |
| O60447 | EVI5 | ecotropic viral integration site 5 |
| Q6ZV73 | Fgd6 | FYVE, RhoGEF and PH domain containing 6 |
| Q8N3X1 | FNBP4 | formin binding protein 4 |
| P85037 | Foxk1 | forkhead box K1 |
| P58012 | Foxl2 | forkhead box L2 |
| Q9UQL6 | HDAC5 | histone deacetylase 5 |
| P37235 | hpcal1 | hippocalcin-like 1 |
| P35475 | Idua | iduronidase, alpha-L- |
| O00370 | LORF2 | LINE-1 retrotransposable element |
| Q5SY16 | NOL9 | nucleolar protein 9 |
| P46531 | notch1 | Notch homolog 1, translocation-associated (Drosophila) |
| Q15738 | nsdhl | NAD(P) dependent steroid dehydrogenase-like |
| Q7Z3B4 | NUP54 | nucleoporin 54kDa |
| O15381 | NVL | nuclear VCP-like |
| Q16825 | PTPN21 | protein tyrosine phosphatase, non-receptor type 21 |
| Q8IZ41 | Rasef | RAS and EF-hand domain containing |
| Q9UHR5 | Sap30bp | SAP30 binding protein |
| Q9HD40 | sepsecs | Sep (O-phosphoserine) tRNA:Sec (selenocysteine) tRNA synthase |
| Q96BD0 | SLCO4A1 | solute carrier organic anion transporter family, member 4A1 |
| Q6ZQN7 | SLCO4C1 | solute carrier organic anion transporter family, member 4C1 |
| Q6Q759 | SPAG17 | sperm associated antigen 17 |
| O95238 | SPDEF | SAM pointed domain containing ets transcription factor |
| Q9H5L6 | THAP9 | THAP domain containing 9 |
| Q86W42 | THOC6 | THO complex 6 homolog (Drosophila) |
| Q53HI1 | UNC50 | unc-50 homolog (C. elegans) |
| Q9Y5J1 | utp18 | UTP18, small subunit (SSU) processome component, homolog (yeast) |
| Q6RFH5 | WDR74 | WD repeat domain 74 |
| Q8N5D0 | WDTC1 | WD and tetratricopeptide repeats 1 |
| Q8N184 | ZNF567 | zinc finger protein 567 |
| Q8IYH5 | ZZZ3 | zinc finger, ZZ-type containing 3 |

**Supplementary Table S4:** Putative annotation of transcripts upregulated in E3 experiment compared to control samples. Lines annotated with yellow, blue and green are those involved in the three main STRING networks identified in these transcripts

| SwissProt ID | Gene Symbol | Gene Name |
|---|---|---|
| P33527 | ABCC1 | ATP-binding cassette, sub-family C (CFTR/MRP), member 1 |
| O00370 | Abcc3 | ATP-binding cassette, sub-family C (CFTR/MRP), member 3 |
| Q8TB40 | abhd4 | abhydrolase domain containing 4 |
| Q9NPJ3 | ACOT13 | acyl-CoA thioesterase 13 |
| Q09666 | ahnak | AHNAK nucleoprotein |
| Q96NU7 | amdhd1 | amidohydrolase domain containing 1 |
| Q99856 | arid3a | AT rich interactive domain 3A (BRIGHT-like) |
| Q9H6L4 | ARMC7 | armadillo repeat containing 7 |
| Q5FYB0 | ARSJ | arylsulfatase family, member J |
| P08243 | asnS | asparagine synthetase |
| P18848 | ATF4 | activating transcription factor 4 (tax-responsive enhancer element |
| O43505 | b3gnt1 | UDP-GlcNAc:betaGal beta-1,3-N-acetylglucosaminyltransferase 1; UDP- |
| Q99933 | bag1 | BCL2-associated athanogene |
| Q9NRL2 | BAZ1A | bromodomain adjacent to zinc finger domain, 1A |
| Q6W2J9 | bcor | BCL6 co-repressor |
| P35226 | BMI1 | BMI1 polycomb ring finger oncogene |
| P12643 | bmp2 | bone morphogenetic protein 2 |
| Q08AG7 | c13orf37 | chromosome 13 open reading frame 37 |
| Q63HM2 | C14orf13 | chromosome 14 open reading frame 135 |
| Q9UJK0 | c16orf42 | chromosome 16 open reading frame 42 |
| Q9BSD7 | C1orf57 | chromosome 1 open reading frame 57; NTPCR |
| Q9NVP4 | C20orf12 | chromosome 20 open reading frame 12 |
| Q9NWY4 | C4orf27 | chromosome 4 open reading frame 27 |
| O43598 | C6orf108 | chromosome 6 open reading frame 108 |
| Q13951 | Cbfb | core-binding factor, beta subunit |
| O75828 | Cbr3 | carbonyl reductase 3 |
| Q9BW85 | CCDC94 | coiled-coil domain containing 94 |
| Q9UEE9 | cfdp1 | craniofacial development protein 1 |
| Q9H2X0 | CHRD | chordin |
| Q9NPF2 | CHST11 | carbohydrate (chondroitin 4) sulfotransferase 11 |
| Q9UJA2 | CRLS1 | cardiolipin synthase 1 |
| Q12996 | CSTF3 | cleavage stimulation factor, 3' pre-RNA, subunit 3, 77kDa |
| Q9UBR2 | CTSZ | cathepsin Z |
| Q9NXE8 | Cwc25 | coiled-coil domain containing 49 |
| Q16678 | CYP1B1 | cytochrome P450, family 1, subfamily B, polypeptide 1 |
| P10635 | cyp2d6 | cytochrome P450, family 2, subfamily D, polypeptide 6 |
| P08684 | CYP3A4 | cytochrome P450, family 3, subfamily A, polypeptide 4 |
| Q99489 | DDO | D-aspartate oxidase |
| Q7L014 | ddx46 | DEAD (Asp-Glu-Ala-Asp) box polypeptide 46 |
| Q9H2U1 | DHX36 | DEAH (Asp-Glu-Ala-His) box polypeptide 36 |
| Q8WXX0 | DNAH7 | dynein, axonemal, heavy chain 7 |
| Q9NZQ0 | DNAJC27 | DnaJ (Hsp40) homolog, subfamily C, member 27 |
| Q9ULA0 | DNPEP | aspartyl aminopeptidase |
| Q9BQC3 | DPH2 | DPH2 homolog (S. cerevisiae) |
| O43854 | edil3 | EGF-like repeats and discoidin I-like domains 3 |
| Q8N9N8 | Eif1ad | eukaryotic translation initiation factor 1A domain containing |
| Q8IUD2 | ERC1 | ELKS/RAB6-interacting/CAST family member 1 |
| Q03828 | EVX2 | even-skipped homeobox 2 |
| Q96CS3 | FAF2 | Fas associated factor family member 2 |
| Q6ZV73 | Fgd6 | FYVE, RhoGEF and PH domain containing 6 |

| | | |
|---|---|---|
| P07954 | FH | fumarate hydratase |
| Q8N8Q3 | flj35220 | hypothetical protein FLJ35220 |
| Q8N3X1 | FNBP4 | formin binding protein 4 |
| P58012 | Foxl2 | forkhead box L2 |
| Q14331 | frg1 | FSHD region gene 1 |
| Q9NXP7 | GIN1 | gypsy retrotransposon integrase 1 |
| P23378 | Gldc | glycine dehydrogenase (decarboxylating) |
| Q13439 | golga4 | golgi autoantigen, golgin subfamily a, 4 |
| Q9UHW5 | Gpn3 | GPN-loop GTPase 3 |
| Q96SL4 | GPX7 | glutathione peroxidase 7 |
| Q16772 | GSTA3 | glutathione S-transferase alpha 3 |
| P78417 | GSTO1 | glutathione S-transferase omega 1 |
| P30711 | GSTT1 | glutathione S-transferase theta 1 |
| Q8N3Z3 | Gtpbp8 | GTP-binding protein 8 (putative) |
| Q96MB7 | HARBI1 | harbinger transposase derived 1 |
| Q7Z4H3 | hddc2 | HD domain containing 2 |
| Q7Z4V5 | Hdgfrp2 | hepatoma-derived growth factor-related protein 2 |
| Q9H0R4 | hdhd2 | haloacid dehalogenase-like hydrolase domain containing 2 |
| P54198 | HIRA | HIR histone cell cycle regulation defective homolog A (S. cerevisia |
| Q99880 | Hist1h2b | histone cluster 1, H2bl |
| P61978 | Hnrnpk | heterogeneous nuclear ribonucleoprotein K; similar to heterogeneous |
| P08107 | HSPA1A | heat shock 70kDa protein 1A; heat shock 70kDa protein 1B |
| O75874 | IDH1 | isocitrate dehydrogenase 1 (NADP+), soluble |
| P35475 | Idua | iduronidase, alpha-L- |
| Q13123 | Ik | similar to CG18005; IK cytokine, down-regulator of HLA II |
| Q96LU5 | IMMP1L | IMP1 inner mitochondrial membrane peptidase-like (S. cerevisiae) |
| P10914 | Irf1 | interferon regulatory factor 1 |
| Q7LBC6 | KDM3B | lysine (K)-specific demethylase 3B |
| Q12767 | KIAA0195 | KIAA0195 |
| Q99538 | LGMN | legumain |
| Q9C099 | LRRCC1 | leucine rich repeat and coiled-coil domain containing 1 |
| O95232 | LUC7L3 | cisplatin resistance-associated overexpressed protein |
| O60502 | mgea5 | meningioma expressed antigen 5 (hyaluronidase) |
| Q5JRA6 | MIA3 | melanoma inhibitory activity family, member 3 |
| P22894 | MMP8 | matrix metallopeptidase 8 (neutrophil collagenase) |
| Q7Z2W9 | MRPL21 | mitochondrial ribosomal protein L21 |
| Q8WY64 | MYLIP | myosin regulatory light chain interacting protein |
| O95251 | Myst2 | MYST histone acetyltransferase 2; KAT7 |
| Q96S42 | NODAL | nodal homolog (mouse) |
| P46531 | notch1 | Notch homolog 1, translocation-associated (Drosophila) |
| Q96CB9 | nsun4 | NOL1/NOP2/Sun domain family, member 4 |
| Q13608 | PEX6 | peroxisomal biogenesis factor 6 |
| Q9P215 | Pogk | pogo transposable element with KRAB domain |
| Q9H9Y6 | Polr1b | polymerase (RNA) I polypeptide B, 128kDa |
| Q9BT43 | Polr3gl | polymerase (RNA) III (DNA directed) polypeptide G (32kD)-like |
| O43172 | PRP4 | U4/U6 small ribonuclear protein PRP4 |
| O15438 | PRPF4 | PRP4 pre-mRNA processing factor 4 homolog (yeast) |
| O94906 | PRPF6 | similar to U5 snRNP-associated 102 kDa protein (U5-102 kDa protein) |
| P23467 | PTPRB | protein tyrosine phosphatase, receptor type, B |
| P15918 | RAG1 | recombination activating gene 1 |
| Q86X10 | RALGAPB | KIAA1219 |
| Q8NHQ8 | rassf8 | Ras association (RalGDS/AF-6) domain family (N-terminal) member 8 |
| P98175 | RBM10 | RNA binding motif protein 10 |
| Q8NDT2 | RBM15B | RNA binding motif protein 15B |
| Q86U06 | RBM23 | RNA binding motif protein 23 |

| | | |
|---|---|---|
| P49756 | RBM25 | RNA binding motif protein 25 |
| Q8NBN7 | RDH13 | retinol dehydrogenase 13 (all-trans/9-cis) |
| Q99496 | rnf2 | ring finger protein 2 |
| A6NKG5 | RTL1 | retrotransposon-like |
| Q14151 | SAFB2 | scaffold attachment factor B2 |
| Q9HD40 | sepsecs | Sep (O-phosphoserine) tRNA:Sec (selenocysteine) tRNA synthase |
| Q9NVD3 | SETD4 | SET domain containing 4 |
| Q12908 | SLC10A2 | solute carrier family 10 (sodium/bile acid cotransporter family), m |
| Q8TF71 | SLC16A10 | solute carrier family 16, member 10 (aromatic amino acid tr |
| Q8IZD6 | SLC22A15 | solute carrier family 22, member 15 |
| Q9Y345 | SLC6A5 | solute carrier family 6 (neurotransmitter transporter, glycine), me |
| Q96BD0 | SLCO4A1 | solute carrier organic anion transporter family, member 4A1 |
| Q9H2Y9 | slco5a1 | solute carrier organic anion transporter family, member 5A1 |
| P18583 | SON | SON DNA binding protein |
| Q06945 | Sox4 | SRY (sex determining region Y)-box 4 |
| O95238 | SPDEF | SAM pointed domain containing ets transcription factor |
| Q96T58 | spen | spen homolog, transcriptional regulator (Drosophila) |
| Q9GZT4 | srr | serine racemase |
| Q9NP77 | ssu72 | SSU72 RNA polymerase II CTD phosphatase homolog (S. cerevisiae) |
| O00267 | supt5h | suppressor of Ty 5 homolog (S. cerevisiae) |
| Q4LDE5 | SVEP1 | sushi, von Willebrand factor type A, EGF and pentraxin domain conta |
| O75528 | TADA3 | transcriptional adaptor 3 (NGG1 homolog, yeast)-like |
| Q7Z7C8 | TAF8 | TAF8 RNA polymerase II, TATA box binding protein (TBP)-associated f |
| Q9NXG2 | THUMPD1 | THUMP domain containing 1 |
| Q9Y584 | TIMM22 | translocase of inner mitochondrial membrane 22 homolog (yeast) |
| Q53FA7 | TP53I3 | tumor protein p53 inducible protein 3 |
| Q6PGP7 | TTC37 | tetratricopeptide repeat domain 37 |
| P07437 | tubB | tubulin, beta; similar to tubulin, beta 5; tubulin, beta pseudogene |
| P55851 | UCP2 | uncoupling protein 2 (mitochondrial, proton carrier) |
| Q8IWX7 | UNC45B | unc-45 homolog B (C. elegans) |
| Q53HI1 | UNC50 | unc-50 homolog (C. elegans) |
| Q5SQQ9 | VAX1 | ventral anterior homeobox 1 |
| P23381 | wars | tryptophanyl-tRNA synthetase |
| Q5VUA4 | ZNF318 | zinc finger protein 318 |
| O94892 | ZNF432 | zinc finger protein 432 |
| Q8N184 | ZNF567 | zinc finger protein 567 |
| Q5TYW1 | ZNF658 | zinc finger protein 658 |

**Supplementary Table S5:** Putative annotation of transcripts upregulated in CE4 experiment compared to control samples. Most of the transcripts cluster in one big network so no STRING networks are annotated for this gene listing

| SwissProt ID | Gene Symbol | Gene Name |
|---|---|---|
| O95477 | ABCA1 | ATP-binding cassette, sub-family A (ABC1), member 1 |
| O15439 | abcc4 | ATP-binding cassette, sub-family C (CFTR/MRP), member 4 |
| Q8IZP0 | Abi1 | abl-interactor 1 |
| Q12979 | ABR | active BCR-related gene |
| Q04771 | acvr1 | activin A receptor, type I |
| P55265 | Adar | adenosine deaminase, RNA-specific |
| O60503 | ADCY9 | adenylate cyclase 9 |
| Q9UPQ3 | AGAP1 | ArfGAP with GTPase domain, ankyrin repeat and PH domain 1 |
| O00468 | AGRN | agrin |
| Q9UPW5 | AGTPBP1 | ATP/GTP binding protein 1 |
| Q8N6S4 | Ankrd13c | ankyrin repeat domain 13C |
| O15084 | ANKRD28 | ankyrin repeat domain 28 |
| P09525 | anxa4 | annexin A4 |
| Q13367 | AP3B2 | adaptor-related protein complex 3, beta 2 subunit |
| Q92572 | ap3s1 | adaptor-related protein complex 3, sigma 1 subunit |
| Q8IW19 | aplf | aprataxin and PNKP like factor |
| P61204 | ARF3 | ADP-ribosylation factor 3 |
| Q9P2F6 | ARHGAP20 | Rho GTPase activating protein 20 |
| Q9UNA1 | arhgap26 | Rho GTPase activating protein 26 |
| P36405 | Arl3 | ADP-ribosylation factor-like 3 |
| Q96B67 | arrdc3 | arrestin domain containing 3 |
| Q6PL18 | ATAD2 | ATPase family, AAA domain containing 2 |
| Q2TAZ0 | Atg2a | ATG2 autophagy related 2 homolog A (S. cerevisiae) |
| Q96BY7 | ATG2B | ATG2 autophagy related 2 homolog B (S. cerevisiae) |
| Q9NT62 | ATG3 | ATG3 autophagy related 3 homolog (S. cerevisiae) |
| P38606 | Atp6v1a | ATPase, H+ transporting, lysosomal 70kDa, V1 subunit A |
| P21283 | ATP6V1C1 | ATPase, H+ transporting, lysosomal 42kDa, V1 subuni |
| O43505 | B3GNT2 | UDP-GlcNAc:betaGal beta-1,3-N-acetylglucosaminyltransferase |
| Q9UBX8 | B4GALT6 | UDP-Gal:betaGlcNAc beta 1,4- galactosyltransferase, polypep |
| O14874 | BCKDK | branched chain ketoacid dehydrogenase kinase |
| P38398 | BRCA1 | breast cancer 1, early onset |
| Q9UID3 | c11orf2 | chromosome 11 open reading frame2 |
| Q9Y4D8 | c12orf51 | chromosome 12 open reading frame 51 |
| Q8IXR9 | C12orf56 | chromosome 12 open reading frame 56 |
| Q96D70 | c19orf22 | chromosome 19 open reading frame 22 |
| Q8WUQ7 | C19orf29 | chromosome 19 open reading frame 29 |
| Q9NVP4 | C20orf12 | chromosome 20 open reading frame 12 |
| Q5NDL2 | c3orf64 | chromosome 3 open reading frame 64 |
| Q96LT7 | C9orf72 | chromosome 9 open reading frame 72 |
| P00918 | CA2 | carbonic anhydrase II |
| Q08289 | CACNB2 | calcium channel, voltage-dependent, beta 2 subunit |
| Q96GE6 | calml4 | calmodulin-like 4 |
| O15484 | CAPN5 | calpain 5 |
| A2VCL2 | CC162 | coiled coil domain containing protein 162 |
| Q9Y592 | CCDC41 | coiled-coil domain containing 41 |
| Q8IWF9 | CCDC83 | coiled-coil domain containing 83 |
| P30260 | cdc27 | cell division cycle 27 homolog (S. cerevisiae) |

| | | |
|---|---|---|
| Q16543 | cdc37 | cell division cycle 37 homolog (S. cerevisiae) |
| P19022 | CDH2 | cadherin 2, type 1, N-cadherin (neuronal) |
| Q8N960 | CEP120 | centrosomal protein 120kDa |
| Q9UQN3 | Chmp2b | chromatin modifying protein 2B |
| Q9BY43 | CHMP4A | chromatin modifying protein 4A |
| Q9H444 | CHMP4B | chromatin modifying protein 4B |
| Q9NZZ3 | Chmp5 | chromatin modifying protein 5 |
| Q14008 | ckap5 | cytoskeleton associated protein 5 |
| O94985 | CLSTN1 | calsyntenin 1 |
| Q8N129 | Cnpy4 | canopy 4 homolog (zebrafish) |
| Q9Y215 | COLQ | collagen-like tail subunit (single strand of homotrimer) of |
| Q9HCH3 | CPNE5 | copine V |
| P50416 | Cpt1a | carnitine palmitoyltransferase 1A (liver) |
| Q5TZA2 | Crocc | ciliary rootlet coiled-coil, rootletin |
| P41240 | CSK | c-src tyrosine kinase |
| Q13948 | CUX1 | cut-like homeobox 1 |
| Q9H720 | CWH43 | hypothetical protein FLJ21511 |
| Q7L1T6 | CYB5R4 | cytochrome b5 reductase 4 |
| Q9NQC7 | CYLD | cylindromatosis (turban tumor syndrome) |
| P20813 | CYP2B6 | cytochrome P450, family 2, subfamily B, polypeptide 6 |
| O95786 | DDX58 | DEAD (Asp-Glu-Ala-Asp) box polypeptide 58 |
| Q8WYQ5 | Dgcr8 | DiGeorge syndrome critical region gene 8 |
| Q8N5I4 | DHRSX | dehydrogenase/reductase (SDR family) X-linked |
| Q96C10 | dhx58 | DEXH (Asp-Glu-X-His) box polypeptide 58 |
| Q05193 | DNM1 | dynamin 1 |
| O00429 | DNM1L | dynamin 1-like |
| P50570 | DNM2 | dynamin 2 |
| Q9NRW4 | dusp22 | similar to mitogen-activated protein kinase phosphatase x; |
| Q16690 | dusp5 | dual specificity phosphatase 5 |
| P42892 | ECE1 | endothelin converting enzyme 1 |
| O14602 | EIF1AY | eukaryotic translation initiation factor 1A, Y-linked |
| P26378 | ELAVL4 | ELAV (embryonic lethal, abnormal vision, Drosophila)-like 4 |
| O94905 | Erlin2 | ER lipid raft associated 2 |
| Q14674 | ESPL1 | extra spindle pole bodies homolog 1 (S. cerevisiae) |
| P14921 | ETS1 | v-ets erythroblastosis virus E26 oncogene homolog 1 (avian) |
| P50549 | etv1 | ets variant 1 |
| Q96A65 | EXOC4 | exocyst complex component 4 |
| Q9UNN5 | FAF1 | Fas (TNFRSF6) associated factor 1 |
| Q9H5Z6 | FAM124B | family with sequence similarity 124B |
| Q8N128 | fam177a1 | family with sequence similarity 177, member A1 |
| Q9GZU8 | fam192a | NEFA-interacting nuclear protein NIP30 |
| Q8TBR7 | FAM57A | family with sequence similarity 57, member A |
| Q5T7M9 | fam69a | family with sequence similarity 69, member A |
| Q5HYJ3 | fam76b | family with sequence similarity 76, member B |
| Q9UKA1 | Fbxl5 | F-box and leucine-rich repeat protein 5 |
| Q9UHY8 | fez2 | fasciculation and elongation protein zeta 2 (zygin II) |
| P21802 | FGFR2 | fibroblast growth factor receptor 2 |
| Q8TF40 | FNIP1 | folliculin interacting protein 1 |
| Q9P2Q2 | Frmd4a | FERM domain containing 4A |
| Q8N475 | Fstl5 | follistatin-like 5 |
| Q99999 | GAL3ST1 | galactose-3-O-sulfotransferase 1 |
| Q96CN9 | gcc1 | GRIP and coiled-coil domain containing 1 |

| | | |
|---|---|---|
| O75603 | GCM2 | glial cells missing homolog 2 (Drosophila) |
| Q9NXN4 | GDAP2 | ganglioside induced differentiation associated protein 2 |
| H0YKK7 | GG6LS | putative golgin subfamily A member 6-like protein 19 |
| Q5VSY0 | Gkap1 | G kinase anchoring protein 1 |
| P60983 | gmfb | glia maturation factor, beta |
| P09471 | GNAO1 | guanine nucleotide binding protein (G protein), alpha activ |
| Q9Y223 | gne | glucosamine (UDP-N-acetyl)-2-epimerase/N-acetylmannosamine |
| Q16774 | GUK1 | guanylate kinase 1 |
| P42357 | hal | histidine ammonia-lyase |
| P31268 | HOXA7 | homeobox A7 |
| Q05084 | Ica1 | islet cell autoantigen 1, 69kDa |
| P46940 | IQGAP1 | IQ motif containing GTPase activating protein 1 |
| Q6NYC1 | JMJD6 | jumonji domain containing 6 |
| Q14678 | KANK1 | KN motif and ankyrin repeat domains 1; similar to ankyrin r |
| Q8IYT4 | KATNAL2 | katanin p60 subunit A-like 2 |
| Q674X7 | kaz | kazrin |
| Q9NS40 | KCNH7 | potassium voltage-gated channel, subfamily H (eag-related), |
| Q6PIL6 | KCNIP4 | Kv channel interacting protein 4 |
| Q14500 | KCNJ18 | similar to hkir2.2x; similar to inward rectifying K+ channe |
| Q9UGI6 | KCNN3 | potassium intermediate/small conductance calcium-activated |
| O15550 | Kdm6a | lysine (K)-specific demethylase 6A |
| O60303 | kiaa0556 | KIAA0556 |
| Q9Y6X3 | KIAA0892 | KIAA0892 |
| Q9HCE0 | kiaa1632 | KIAA1632 |
| Q9BVG8 | Kifc3 | kinesin family member C3 |
| Q9NSK0 | KLC4 | kinesin light chain 4 |
| O00629 | KPNA4 | karyopherin alpha 4 (importin alpha 3) |
| Q9H9P8 | L2HGDH | L-2-hydroxyglutarate dehydrogenase |
| P00338 | LdhA | lactate dehydrogenase A |
| O43766 | LIAS | lipoic acid synthetase |
| Q9Y561 | LRP12 | low density lipoprotein-related protein 12 |
| O75581 | LRP6 | low density lipoprotein receptor-related protein 6 |
| Q86X45 | LRRC6 | leucine rich repeat containing 6 |
| Q9HD34 | LYRM4 | LYR motif containing 4 |
| O15525 | MAFG | v-maf musculoaponeurotic fibrosarcoma oncogene homolog G |
| Q9UNF1 | MAGED2 | melanoma antigen family D, 2 |
| Q16706 | MAN2A1 | mannosidase, alpha, class 2A, member 1 |
| P55145 | manf | mesencephalic astrocyte-derived neurotrophic factor |
| Q99759 | MAP3K3 | mitogen-activated protein kinase kinase kinase 3 |
| O43318 | MAP3K7 | mitogen-activated protein kinase kinase kinase 7 |
| P28482 | MAPK1 | mitogen-activated protein kinase 1 |
| Q8IW41 | MAPKAPK5 | mitogen-activated protein kinase-activated protein |
| P10636 | mapt | microtubule-associated protein tau |
| P23508 | MCC | mutated in colorectal cancers |
| Q7L590 | Mcm10 | minichromosome maintenance complex component 10 |
| Q9HAF1 | MEAF6 | chromosome 1 open reading frame 149 |
| Q9BV79 | MECR | mitochondrial trans-2-enoyl-CoA reductase |
| Q14696 | MESDC2 | mesoderm development candidate 2 |
| Q08431 | MFGE8 | milk fat globule-EGF factor 8 protein |
| Q09328 | Mgat5 | mannosyl (alpha-1,6-)-glycoprotein beta-1,6-N-acetyl-glucos |
| Q8N119 | MMP21 | matrix metallopeptidase 21 |
| Q9Y5R2 | mmp24 | matrix metallopeptidase 24 (membrane-inserted) |

| | | |
|---|---|---|
| P51948 | Mnat1 | menage a trois homolog 1, cyclin H assembly factor (Xenopus |
| Q9NZB8 | Mocs1 | molybdenum cofactor synthesis 1 |
| O15442 | MPPED1 | metallophosphoesterase domain containing 1 |
| Q5JR59 | Mtus2 | KIAA0774 |
| Q15746 | MYLK | myosin light chain kinase |
| O95544 | nadK | NAD kinase |
| P54920 | napA | N-ethylmaleimide-sensitive factor attachment protein, alpha |
| Q96PU5 | Nedd4l | neural precursor cell expressed, developmentally down-regul |
| Q96PY6 | nek1 | NIMA (never in mitosis gene a)-related kinase 1 |
| P21359 | NF1 | neurofibromin 1 |
| Q15270 | NKX1-1 | NK1 homeobox 1 |
| P43699 | nkx2-1 | NK2 homeobox 1 |
| Q96PH1 | nox5 | NADPH oxidase, EF-hand calcium binding domain 5 |
| P01111 | NRAS | neuroblastoma RAS viral (v-ras) oncogene homolog |
| Q9BW91 | nudt9 | nudix (nucleoside diphosphate linked moiety X)-type motif 9 |
| Q9NX40 | ociad1 | OCIA domain containing 1 |
| O95897 | olfm2 | olfactomedin 2 |
| O60313 | Opa1 | optic atrophy 1 (autosomal dominant) |
| Q92882 | ostf1 | osteoclast stimulating factor 1 |
| Q6ZNF0 | papL | iron/zinc purple acid phosphatase-like protein |
| Q9BYG4 | PARD6G | par-6 partitioning defective 6 homolog gamma (C. elegans) |
| O14737 | PDCD5 | programmed cell death 5 |
| Q9P0J1 | Pdp1 | pyruvate dehyrogenase phosphatase catalytic subunit 1 |
| Q6ZMN7 | Pdzrn4 | PDZ domain containing ring finger 4 |
| P08237 | PFKM | phosphofructokinase, muscle |
| O15173 | PGRMC2 | progesterone receptor membrane component 2 |
| Q96QT6 | PHF12 | PHD finger protein 12 |
| Q9BUL5 | Phf23 | PHD finger protein 23 |
| Q9UPP1 | PHF8 | PHD finger protein 8 |
| P42338 | Pik3cb | phosphoinositide-3-kinase, catalytic, beta polypeptide |
| P27986 | Pik3r1 | phosphoinositide-3-kinase, regulatory subunit 1 (alpha) |
| Q9Y2I7 | Pikfyve | phosphoinositide kinase, FYVE finger containing |
| P48426 | PIP4K2A | phosphatidylinositol-5-phosphate 4-kinase, type II, alpha |
| Q13393 | PLD1 | phospholipase D1, phosphatidylcholine-specific |
| Q9H8W4 | plekhf2 | pleckstrin homology domain containing, family F (with FYVE |
| A6NEE1 | PLHD1 | pleckstrin homology domain containing family D member 1 |
| O15162 | plscr1 | phospholipid scramblase 1 |
| O75051 | Plxna2 | plexin A2 |
| P51805 | Plxna3 | plexin A3 |
| P06746 | polB | polymerase (DNA directed), beta |
| Q9HCU8 | POLD4 | polymerase (DNA-directed), delta 4 |
| Q9NRF9 | POLE3 | polymerase (DNA directed), epsilon 3 (p17 subunit) |
| Q03181 | Ppard | peroxisome proliferator-activated receptor delta |
| Q16537 | PPP2R5E | protein phosphatase 2, regulatory subunit B', epsilon isofo |
| O43663 | prc1 | protein regulator of cytokinesis 1 |
| Q05655 | PRKCD | protein kinase C, delta |
| Q96LA8 | PRMT6 | protein arginine methyltransferase 6 |
| Q14997 | Psme4 | proteasome (prosome, macropain) activator subunit 4 |
| O43586 | PSTPIP1 | proline-serine-threonine phosphatase interacting protein 1 |
| Q8N8N7 | PTGR2 | prostaglandin reductase 2 |
| P43378 | Ptpn9 | protein tyrosine phosphatase, non-receptor type 9 |
| Q16849 | PTPRN | protein tyrosine phosphatase, receptor type, N |

| | | |
|---|---|---|
| Q9UMZ3 | ptprq | protein tyrosine phosphatase, receptor type, Q |
| P61026 | RAB10 | RAB10, member RAS oncogene family |
| P59190 | RAB15 | RAB15, member RAS onocogene family |
| Q9NP72 | RAB18 | RAB18, member RAS oncogene family |
| P62820 | RAB1A | RAB1A, member RAS oncogene family |
| Q9UL25 | rab21 | RAB21, member RAS oncogene family |
| Q969Q5 | RAB24 | RAB24, member RAS oncogene family |
| P51148 | rab5c | RAB5C, member RAS oncogene family |
| Q92930 | RAB8B | RAB8B, member RAS oncogene family |
| P11233 | RALA | v-ral simian leukemia viral oncogene homolog A (ras related |
| Q9UJF2 | RASAL2 | RAS protein activator like 2 |
| Q0VAM2 | RASGEF1B | RasGEF domain family, member 1B |
| P50749 | RASSF2 | Ras association (RalGDS/AF-6) domain family member 2 |
| Q8WWW0 | rassf5 | Ras association (RalGDS/AF-6) domain family member 5 |
| Q14206 | RCAN2 | regulator of calcineurin 2 |
| P35241 | rdx | radixin |
| P61586 | Rhoa | ras homolog gene family, member A |
| Q9UQ26 | RIMS2 | regulating synaptic membrane exocytosis 2 |
| Q96PX1 | RNF157 | ring finger protein 157 |
| Q8IYW5 | rnf168 | ring finger protein 168 |
| Q96GF1 | RNF185 | ring finger protein 185 |
| Q99496 | rnf2 | ring finger protein 2 |
| Q96LT9 | RNPC3 | RNA-binding region (RNP1, RRM) containing 3 |
| O75582 | RPS6KA5 | ribosomal protein S6 kinase, 90kDa, polypeptide 5 |
| Q8WWV3 | RTN4IP1 | reticulon 4 interacting protein 1 |
| Q9HBX9 | RXFP1 | relaxin/insulin-like family peptide receptor 1 |
| Q8WVM8 | SCFD1 | sec1 family domain containing 1 |
| O76054 | Sec14l2 | SEC14-like 2 (S. cerevisiae) |
| O43236 | SEPT4 | septin 4 |
| Q16181 | SEPT7 | septin 7 |
| Q9NZV6 | SEPX1 | selenoprotein X, 1 |
| Q96HU1 | SGSM3 | small G protein signaling modulator 3 |
| P29353 | SHC1 | SHC (Src homology 2 domain containing) transforming protein |
| Q8IXJ6 | Sirt2 | sirtuin (silent mating type information regulation 2 homolo |
| O75563 | skap2 | src kinase associated phosphoprotein 2 |
| Q16348 | Slc15a2 | solute carrier family 15 (H+/peptide transporter), member 2 |
| Q9Y226 | SLC22A13 | solute carrier family 22 (organic anion transporter |
| Q8NEW0 | SLC30A7 | solute carrier family 30 (zinc transporter), member 7 |
| Q8IWA5 | SLC44A2 | solute carrier family 44, member 2 |
| Q9Y2W3 | Slc45a1 | solute carrier family 45, member 1 |
| Q96NT5 | Slc46a1 | solute carrier family 46 (folate transporter), member 1 |
| Q2M3M2 | Slc5a9 | solute carrier family 5 (sodium/glucose cotransporter), mem |
| Q9UN76 | Slc6a14 | solute carrier family 6 (amino acid transporter), member 14 |
| Q9H2G2 | SLK | STE20-like kinase (yeast) |
| Q9UPR3 | smg5 | Smg-5 homolog, nonsense mediated mRNA decay factor (C. elegans) |
| Q86US8 | SMG6 | Smg-6 homolog, nonsense mediated mRNA decay factor (C. eleg |
| Q9H4F8 | SMOC1 | SPARC related modular calcium binding 1 |
| P53814 | smtn | smoothelin |
| P60880 | SNAP25 | synaptosomal-associated protein, 25kDa |
| Q9NRS6 | snx15 | sorting nexin 15 |
| Q6X4U4 | SOSTDC1 | sclerostin domain containing 1 |
| Q02446 | SP4 | Sp4 transcription factor |

| | | |
|---|---|---|
| O43791 | SPOP | speckle-type POZ protein |
| Q9GZT4 | srr | serine racemase |
| Q8WVM7 | STAG1 | stromal antigen 1 |
| Q92783 | stam | signal transducing adaptor molecule (SH3 domain and ITAM mo |
| Q9Y2H1 | stk38l | serine/threonine kinase 38 like |
| P61764 | STXBP1 | syntaxin binding protein 1 |
| Q9H7L9 | suds3 | suppressor of defective silencing 3 homolog (S. cerevisiae) |
| Q9H5I1 | suv39h2 | suppressor of variegation 3-9 homolog 2 (Drosophila) |
| O95926 | Syf2 | SYF2 homolog, RNA splicing factor (S. cerevisiae) |
| P57105 | synj2bp | synaptojanin 2 binding protein |
| Q86SS6 | SYT9 | synaptotagmin IX |
| Q96C24 | SYTL4 | synaptotagmin-like 4 |
| Q5VWG9 | taf3 | TAF3 RNA polymerase II, TATA box binding protein (TBP)-asso |
| Q9Y6I9 | Tex264 | testis expressed 264 |
| Q92481 | TFAP2B | transcription factor AP-2 beta (activating enhancer binding |
| Q96J01 | THOC3 | similar to THO complex 3; THO complex 3 |
| Q8NBT3 | Tmem145 | transmembrane protein 145 |
| P82094 | TMF1 | TATA element modulatory factor 1 |
| P28289 | TMOD1 | tropomodulin 1 |
| Q8IUR5 | TMTC1 | transmembrane and tetratricopeptide repeat containing 1 |
| P55327 | TPD52 | tumor protein D52 |
| Q86SZ2 | TRAPPC6B | trafficking protein particle complex 6B |
| P36406 | trim23 | tripartite motif-containing 23 |
| Q13507 | Trpc3 | transient receptor potential cation channel, subfamily C, m |
| Q8TEL6 | TRPC4AP | transient receptor potential cation channel, subfamily C, m |
| Q8NG11 | TSPAN14 | tetraspanin 14 |
| Q9BWV7 | TTLL2 | tubulin tyrosine ligase-like family, member 2 |
| Q8WZ42 | TTN | titin |
| O95551 | Ttrap | TRAF and TNF receptor associated protein |
| Q8TDH9 | TXNDC5 | thioredoxin domain containing 5 (endoplasmic reticulum); mu |
| Q9Y2X8 | ube2d4 | ubiquitin-conjugating enzyme E2D 4 (putative) |
| P62256 | Ube2h | ubiquitin-conjugating enzyme E2H (UBC8 homolog, yeast) |
| P61086 | UBE2K | ubiquitin-conjugating enzyme E2K (UBC1 homolog, yeast) |
| Q712K3 | ube2r2 | ubiquitin-conjugating enzyme E2R 2 |
| Q13404 | Ube2v1 | ubiquitin-conjugating enzyme E2 variant 1; ubiquitin-conjug |
| Q8IWX7 | UNC45B | unc-45 homolog B (C. elegans) |
| O75445 | USH2A | Usher syndrome 2A (autosomal recessive, mild) |
| Q9Y6I4 | usp3 | ubiquitin specific peptidase 3 |
| Q96K76 | usp47 | ubiquitin specific peptidase 47 |
| Q93009 | Usp7 | ubiquitin specific peptidase 7 (herpes virus-associated) |
| Q9H269 | VPS16 | vacuolar protein sorting 16 homolog A (S. cerevisiae) |
| Q96AX1 | vps33a | vacuolar protein sorting 33 homolog A (S. cerevisiae) |
| O75351 | VPS4B | vacuolar protein sorting 4 homolog B (S. cerevisiae) |
| Q9BZH6 | WDR11 | bromodomain and WD repeat domain containing 2 |
| Q8NEZ3 | WDR19 | WD repeat domain 19 |
| Q9P2L0 | WDR35 | WD repeat domain 35 |
| Q9Y2I8 | WDR37 | WD repeat domain 37 |
| Q9Y4E6 | WDR7 | WD repeat domain 7 |
| P41221 | WNT5A | wingless-type MMTV integration site family, member 5A |
| P47989 | XDH | xanthine dehydrogenase |
| Q9UBH6 | XPR1 | xenotropic and polytropic retrovirus receptor |
| Q9Y2Z4 | Yars2 | tyrosyl-tRNA synthetase 2, mitochondrial |

| | | |
|---|---|---|
| O15498 | YKT6 | YKT6 v-SNARE homolog (S. cerevisiae) |
| P62699 | YPEL5 | yippee-like 5 (Drosophila) |
| Q9HBF4 | ZFYVE1 | zinc finger, FYVE domain containing 1 |
| Q9C0D3 | ZYG11B | zyg-11 homolog B (C. elegans) |

**Supplementary Table S6:** Putative annotation of transcripts upregulated in E4 experiment compared to control samples. Most transcripts cluster in one big network so no STRING networks are annotated in this gene listing

| SwissProt ID | Gene Symbol | Gene Name |
|---|---|---|
| O95477 | ABCA1 | ATP-binding cassette, sub-family A (ABC1), member 1 |
| O15439 | abcc4 | ATP-binding cassette, sub-family C (CFTR/MRP), member 4 |
| O60706 | abcc9 | ATP-binding cassette, sub-family C (CFTR/MRP), member 9 |
| Q12979 | ABR | active BCR-related gene |
| P28330 | acadl | acyl-Coenzyme A dehydrogenase, long chain |
| Q9H3P7 | ACBD3 | acyl-Coenzyme A binding domain containing 3 |
| O14734 | ACOT8 | acyl-CoA thioesterase 8 |
| Q96GR2 | Acsbg1 | acyl-CoA synthetase bubblegum family member 1 |
| Q9NUB1 | acss1 | acyl-CoA synthetase short-chain family member 1 |
| P68133 | ACTA1 | actin, alpha 1, skeletal muscle |
| Q562R1 | Actbl2 | actin, beta-like 2 |
| P00813 | ada | adenosine deaminase |
| P55265 | Adar | adenosine deaminase, RNA-specific |
| O60503 | ADCY9 | adenylate cyclase 9 |
| Q96SZ5 | Ado | 2-aminoethanethiol (cysteamine) dioxygenase |
| Q9Y4W6 | AFG3L2 | AFG3 ATPase family gene 3-like 2 (yeast) |
| Q8NEM8 | Agbl3 | ATP/GTP binding protein-like 3 |
| O00468 | AGRN | agrin |
| Q9BRQ8 | aifm2 | apoptosis-inducing factor, mitochondrion-associated, 2 |
| Q96NN9 | aifm3 | apoptosis-inducing factor, mitochondrion-associated, 3 |
| P00568 | ak1 | adenylate kinase 1 |
| P13716 | Alad | aminolevulinate, delta-, dehydratase |
| P05091 | ALDH2 | aldehyde dehydrogenase 2 family (mitochondrial) |
| Q02252 | Aldh6a1 | aldehyde dehydrogenase 6 family, member A1 |
| P49419 | ALDH7A1 | aldehyde dehydrogenase 7 family, member A1 |
| P04075 | ALDOA | aldolase A, fructose-bisphosphate |
| P09972 | aldoc | aldolase C, fructose-bisphosphate |
| P18054 | ALOX12 | arachidonate 12-lipoxygenase |
| P09917 | ALOX5 | arachidonate 5-lipoxygenase |
| Q96NU7 | amdhd1 | amidohydrolase domain containing 1 |
| Q9BS18 | ANAPC13 | anaphase promoting complex subunit 13 |
| Q9UJX3 | anapc7 | anaphase promoting complex subunit 7 |
| Q7Z5J8 | ankar | ankyrin and armadillo repeat containing |
| Q9UPS8 | ANKRD26 | ankyrin repeat domain 26 |
| O15084 | ANKRD28 | ankyrin repeat domain 28 |
| Q5TZF3 | ANKRD45 | ankyrin repeat domain 45 |
| Q9NQW6 | Anln | anillin, actin binding protein |
| Q9UPM8 | Ap4e1 | adaptor-related protein complex 4, epsilon 1 subunit |
| Q8IW19 | aplf | aprataxin and PNKP like factor |
| Q8WZ64 | Arap2 | ArfGAP with RhoGAP domain, ankyrin repeat and PH domain 2 |
| P84077 | Arf1 | ADP-ribosylation factor 1 |
| P61204 | ARF3 | ADP-ribosylation factor 3 |
| Q9P2F6 | ARHGAP20 | Rho GTPase activating protein 20 |
| Q15052 | ARHGEF6 | Rac/Cdc42 guanine nucleotide exchange factor (GEF) 6 |
| Q9Y4X5 | ARIH1 | ariadne homolog, ubiquitin-conjugating enzyme E2 binding protein, 1 (Drosophila) |
| Q8NEN0 | ARMC2 | armadillo repeat containing 2 |
| Q96QS3 | ARX | aristaless related homeobox |
| Q8IZT6 | ASPM | asp (abnormal spindle) homolog, microcephaly associated (Drosophila) |
| O94817 | Atg12 | ATG12 autophagy related 12 homolog (S. cerevisiae) |
| Q2TAZ0 | Atg2a | ATG2 autophagy related 2 homolog A (S. cerevisiae) |
| O75366 | AVIL | advillin |
| Q9Y5Z6 | B3galt1 | UDP-Gal:betaGlcNAc beta 1,3-galactosyltransferase, polypeptide 1 |
| O43505 | b3gnt1 | UDP-GlcNAc:betaGal beta-1,3-N-acetylglucosaminyltransferase 1; UDP-GlcNAc:betaGal beta-1,3-N-acetylgluc |
| Q99933 | bag1 | BCL2-associated athanogene |
| Q8TBE0 | BAHD1 | bromo adjacent homology domain containing 1 |
| O75531 | BANF1 | similar to barrier-to-autointegration factor; barrier to autointegration factor 1 |
| Q07812 | BAX | BCL2-associated X protein |
| Q9H6U6 | BCAS3 | breast carcinoma amplified sequence 3 |
| O14874 | BCKDK | branched chain ketoacid dehydrogenase kinase |
| Q07817 | BCL2L1 | BCL2-like 1 |
| P12643 | bmp2 | bone morphogenetic protein 2 |
| Q12981 | BNIP1 | BCL2/adenovirus E1B 19kDa interacting protein 1 |
| P38398 | BRCA1 | breast cancer 1, early onset |

**Supplementary Table S7:** Primer sequences used in Q-PCR analysis

| Contig identifier | Primer sequences[a] | Amplicon size (bp) | Samples | Amplification efficiency | $R^2$ |
|---|---|---|---|---|---|
| 13435 | F: 5'-ACCGACCTTGACATGACAGT-3'<br>R: 5'-AGAAACCCCTGGAGAAGCTC-3' | 191 | Larvae[b] | Day 2: 98%<br>Day 4: 128% | 0.98 |
| | | | Adults | 125% | 0.99 |
| 37720 | F: 5'-TCGCACTGCGGCTGAACGA-3'<br>R: 5'-CATCTTTGCATCAAGAATCGCC-3' | 190 | Larvae | 101% | 0.99 |
| | | | Adults | 112% | 0.99 |
| 44027 | F: 5'-CTTCAAGTGGAGATGCGTGG-3'<br>R: 5'-CAATCCTGCAATCTGACCGG-3' | 203 | Larvae | 102% | 0.99 |
| | | | Adults | 80% | 0.99 |
| 46238 | F: 5'-GACCATCAACTCCTCCACCA-3'<br>R: 5'-GGAATGCGACTTGATCCACC-3' | 207 | Larvae | 99% | 0.98 |
| | | | Adults | 119% | 0.96 |
| 51464 | F: 5'-GGGCTAGTAAGACGGAGACC-3'<br>R: 5'-TCTGTGATGTCCTTGGGCAT-3' | 217 | Larvae | 95% | 0.99 |
| | | | Adults | 100% | 0.97 |
| 52063 | F: 5'-GACCCTTATCGACACGGGAA-3'<br>R: 5'-AGGCGGAGTAGAAACCAACA-3' | 158 | Larvae | 91% | 0.98 |
| | | | Adults | 109% | 0.99 |
| 62178 | F: 5'-TCCACAGCTGCAGATAACCA-3'<br>R: 5'-CTTGTCTTCGGCGCTTACTC-3' | 195 | Larvae | 105% | 0.99 |
| | | | Adults | 99% | 0.99 |
| 62830 | F: 5'-CATCACTGCTAACAAAGAGCA-3'<br>R: 5'-ATTTGGAGTCACTGAGAGCTT-3' | 218 | Larvae | 104% | 0.99 |
| | | | Adults | 111% | 0.99 |
| 62842 | F: 5'-ACAAGAAGGACATCTCTACCA-3'<br>R: 5'-TCCCTAGCTTGGAGTCGTTG-3' | 236 | Larvae | 115% | 0.99 |
| | | | Adults | 101% | 0.99 |
| 18s rRNA | F: 5'-AGTTGGATCTTGGGTCCAGG-3'<br>R: 5'-CCAACGCAATAGAACCGAGG-3' | 209 | Larvae | 111% | 0.99 |
| | | | Adults | 96% | 0.99 |

[a] F: forward primer; R: reverse primer.

[b] A different Day 4 samples-enriched pool was used for Day 4 samples (hence the different efficiency) in order to include one outlier whose Cq were slightly outside the dynamic range of the standard curve prepared with the pool comprising all larval samples.

**Supplementary Table S8:** Raw Cqs of the reference gene (*18S*) used in the qPCR data analysis.

| Developmental stage | Treatment | Sample | Raw Cq |
|---|---|---|---|
| Adults | Control | 1 | 12.06673 |
| | | 2 | 12.82645 |
| | | 3 | 11.37784 |
| | | 4 | 11.80275 |
| | | 5 | 12.36306 |
| | Heat shock | 6 | 11.49351 |
| | | 7 | 12.60066 |
| | | 8 | 12.08099 |
| | | 9 | 12.33766 |
| | | 10 | 11.9397 |
| 2 dpf larvae | Control | 1 | 19.19702 |
| | | 2 | 18.51549 |
| | | 3 | 19.74385 |
| | Heat shock | 4 | 17.96737 |
| | | 5 | 17.41903 |
| | | 6 | 18.91465 |
| 4 dpf larvae | Control | 1 | 10.68156 |
| | | 2 | 14.73164 |
| | | 3 | 15.18901 |
| | Heat shock | 4 | 10.74604 |
| | | 5 | 14.69566 |
| | | 6 | 15.74854 |
